# Immunomodulatory Effects and Mechanisms of *Curcuma* Species and Their Bioactive Compounds: A Review

**DOI:** 10.3389/fphar.2021.643119

**Published:** 2021-04-30

**Authors:** Yuandani, Ibrahim Jantan, Ade Sri Rohani, Imam Bagus Sumantri

**Affiliations:** ^1^ Department of Pharmacology, Faculty of Pharmacy, Universitas Sumatera Utara, Medan, Indonesia; ^2^ Institute of Systems Biology, Universiti Kebangsaan Malaysia, Selangor, Malaysia; ^3^ Department of Pharmaceutical Biology, Faculty of Pharmacy, Universitas Sumatera Utara, Medan, Indonesia

**Keywords:** curcuma species, ethnopharmacology, phytochemicals, immunomodulation, immune system

## Abstract

*Curcuma* species (family: Zingiberaceae) are widely utilized in traditional medicine to treat diverse immune-related disorders. There have been many scientific studies on their immunomodulating effects to support their ethnopharmacological uses. In this review, the efficacy of six *Curcuma* species, namely, *C. longa* L., *C. zanthorrhiza* Roxb*.*, *C. mangga* Valeton & Zijp, *C. aeruginosa* Roxb. *C. zedoaria* (Christm.) Roscoe, and *C. amada* Roxb., and their bioactive metabolites to modulate the immune system, their mechanistic effects, and their potential to be developed into effective and safe immunomodulatory agents are highlighted. Literature search has been carried out extensively to gather significant findings on immunomodulating activities of these plants. The immunomodulatory effects of *Curcuma* species were critically analyzed, and future research strategies and appropriate perspectives on the plants as source of new immunomodulators were discussed. Most of the pharmacological investigations to evaluate their immunomodulatory effects were *in vivo* and *in vitro* experiments on the crude extracts of the plants. The extracts were not chemically characterized or standardized. Of all the *Curcuma* species investigated, the immunomodulatory effects of *C. longa* were the most studied. Most of the bioactive metabolites responsible for the immunomodulating activities were not determined, and mechanistic studies to understand the underlying mechanisms were scanty. There are limited clinical studies to confirm their efficacy in human. Of all the bioactive metabolites, only curcumin is undergoing extensive clinical trials based on its anti-inflammatory properties and main use as an adjuvant for the treatment of cancer. More in-depth studies to understand the underlying mechanisms using experimental *in vivo* animal models of immune-related disorders and elaborate bioavailability, preclinical pharmacokinetics, and toxicity studies are required before clinical trials can be pursued for development into immunomodulatory agents.

## Introduction

The human body has a remarkably sophisticated immune system consisting of white blood cells and specialized immune molecules that protect the body against invading pathogens (Tan and Vanitha, 2004). The immune system is made up of innate and adaptive immune immunity. Innate immunity provides first protection against pathogens, and then it will stimulate adaptive immunity to enhance the protection. Innate immunity is the most rapidly acting immunity. It mostly depends on neutrophils, macrophages, dendritic cells, and monocytes, while T and B cells are involved in adaptive immunity (Beutler, 2004; Saroj et al., 2012). In response to pathogens, leukocytes perform a number of phagocytic activities, including chemotaxis, leukocytes adhesion to vascular endothelial cells, and pathogen engulfment, followed by intracellular killing to eliminate the pathogens (Beutler, 2004; Kobayashi et al., 2005). Phagocytes migrate toward the chemoattractants such as complement (C3a and C3b) and formyl methionyl-leucyl-phenylalanine (fMLP) (a bacterial product) (Luster, 2001). Chemoattractants utilize a similar signal transduction system, namely, G protein–coupled receptor, that is, platelet-activating factor receptor (PAFR), formyl-methionyl-leucyl-phenylalanine receptor (fMLPR), and complement C5a receptor (C5aR). The interaction of chemotactic factor and its receptor stimulates cytoskeletal reorganization, calcium mobilization, and degranulation in heterologous cell types (Firtel and Chung, 2000). The adhesion of leukocytes to vascular endothelial cells is initiated by selection interaction, followed by the interaction of leukocyte integrin of the CD18 complex on the surface of phagocytes with adhesion molecule on endothelial cells (Beutler, 2004). Phagocytosis of microorganism triggers superoxide radical (O_2_^-^) generation and release of reactive oxygen species (ROS) such as hydroxyl radical, hypochlorous acid (HOCl), and chloramines through the activity of myeloperoxidase (MPO). Besides, macrophages are involved in the release of nitric oxide (NO^.^) by inducible nitric oxide synthase (iNOS) (Bogdan, 2001).

Macrophages also modulate adaptive immunity by presenting antigen to CD4 T cells through major histocompatibility complex (MHC) class II antigen. CD4 T cells perform their functions by four subpopulations, which include Th-1, Th-2, Th-17, and CD4 T regulatory (Treg) cells (Chapel et al., 2006). Th cells help B cells develop into plasma cells which can produce antibody and also activate T cells to become activated cytotoxic T cells (Beutler, 2004; Luckheeram et al., 2011). Several cytokines also play essential roles in immune response, which consist of pro-inflammatory cytokines such as tumor necrosis factor-alpha (TNF-α), interleukin 1 (IL-1), IL-6, IL-11, IL-8, and anti-inflammatory cytokines or cytokines inhibitor such as IL-4, IL-10, and IL-13. Cytokines as intercellular messenger molecules have several functions, and these include stimulating phagocyte migration and coordinating early responses of monocytes, macrophages, dendritic cells, and lymphocytes during inflammatory states (Shaikh, 2011). The release of pro-inflammatory cytokines is regulated by nuclear factor-kappa B (NF-ĸB) and mitogen-activated protein kinase (MAPK) pathways (Beyaert et al., 2013). Defects or malfunctions in the immune system can cause disorders of the immune system. Inappropriate reaction to self-antigen is known as autoimmunity such as myasthenia gravis, type 1 diabetes (T1D), systemic lupus erythematosus, Graves’ disease, celiac disease, pernicious anemia, rheumatoid arthritis, and multiple sclerosis. Overactive immune response is known as hypersensitivity reactions, while ineffective immune response is known as immunodeficiency (Zhernakova et al., 2009; Warrington et al., 2011; Beyaert et al., 2013). The diseases which cause the body's immune system to attack the small intestine has affected 1 in 133 people in the United States (Rattue, 2012). A review on incidence and prevalence of Crohn’s disease in several countries reported a gradual increase in incidence and prevalence of this disease. In Malaysia, a study during 2001–2003 showed an increase of prevalence especially among Indians, compared to Chinese and Malay populations. Meanwhile, in Singapore, a study showed that majority patients were Chinese, and there was a trend of increased of prevalence (Economou et al., 2009). Therefore, modulation of the immune response is required in the management and treatment of diseases due to immune system dysfunction (Geetha et al., 2005).

The treatment of inflammatory and immune-related diseases due to defects or disorders of the immune system necessitates modulation of the immune response. Immunomodulation is the process of modifying an immune response by administration of a drug or compound, while immunomodulators are substances which are used to modulate the components of the immune system (Patil et al., 2012). There are several chemical immunomodulators available in the market, that is, prednisone, hydrocortisone, and dexamethasone, which have been used to treat numerous inflammatory diseases. Recombinant proteins have emerged as one important drug to treat cancer, immunodeficiency, and infectious diseases. Cyclosporin A, a microbial peptide, is the most widely used immunosuppressant in transplant rejection treatment (Elgert, 2009). Unfortunately, most of these commercial drugs have side effects. Gastric and intestinal mucosal damage are the commonest adverse effects of NSAIDS. Corticosteroids, an immunosuppressive drug, show various side effects, such as reduced bone marrow and increased skin fragility. Cyclosporin A exhibited toxicities and side effects including nephrotoxic activity and gingival hyperthrophy. Therefore, safer and more effective drugs are required as alternatives. Natural products remain one of the important sources of new and safe anti-inflammatory agents (Elgert, 2009).

In an effort to investigate for safer drugs, ethnopharmacological information can be used to provide preliminary data in the search for new drugs. It can be an indicator of pharmacological activity of natural products that could be further investigated for their mechanisms of action in cellular, animal, and human studies (Flores, 2017). Among them, some therapeutic activities of plant extracts or compounds have been proposed to be due to their effects on the immune system. Many plants of the genus *Curcuma*, especially *C. longa*, *C. zanthorrhiza*, *C. amada*, *C. mangga*, *C. aeruginosa*, and *C. zedoaria*, were reported to modulate the immune functions and possessed a variety of immunomodulatory effects. The strong immunomodulatory activity of these plants was due to their bioactive compounds as their main constituents. Curcuminoids, particularly curcumin, have been reported as the major components of plants in *Curcuma* species. Besides, other compounds, such as xanthorrhizol, have been reported to be present in other *Curcuma* species. A number of reviews on the phytochemistry, and biological and pharmacological activities of the genus *Curcuma* have been published recently (Rajkumari and Sanatombi, 2017; Sun et al., 2017; Dosoki and Setzer, 2018; Chanda and Ramachandra, 2019; Kaliyadasa and Samarasinghe, 2019; Kavitha and Mahadevi, 2020; USDA, 2021). However, there is either no or little and unconcise reports on the immunomodulatory effects of genus *Curcuma* and their bioactive molecules in these articles. In this review, we elaborated the ability of *C. longa* L*.*, *C. zanthorrhiza* Roxb*.*, *C. mangga* Valeton & Zijp, *C. aeruginosa* Roxb*.*, *C. zedoaria* (Christm.) Roscoe, *and C. amada* Roxb*.* and their bioactive metabolites to modulate the immune response in different lineages of the immune system.

## Methods

This comprehensive review was based on updated scientific databases on six major *Curcuma* species, namely, *C. longa*, *C. zanthorrhiza*, *C. manga*, *C. aeruginosa*, *C. zedoaria*, and *C. amada*. Databases were scanned from January 2000 until December 2020 for animal, *in vitro*, and clinical studies. A systematic search of databases with the use of the keywords “curcuma AND immune system,” “curcumin AND immune system,” and each species of *Curcuma* genus, such as “*Curcuma mangga* AND immune system,” “*Curcuma longa* AND immune system,” was carried out. Only published data were included in this study; meanwhile, references without title in English were excluded. Literature search has been carried out extensively to gather data, involving use of published scientific reports in Frontiers, the Science Direct, Scopus, Google Scholar, the Institute for Scientific Information (ISI)-Web of Science, Pub Med, Wiley Online Library, Elsevier, Springer, Taylor and Francis, ACS Publications Today, and other references over the past two decades. The gathered data on the immunomodulating effects of the *Curcuma* species were critically analyzed, and future strategies and appropriate perspectives for the plants as a source of new natural immunomodulators were discussed.

## Taxonomy and Distribution

**FIGURE 1:**
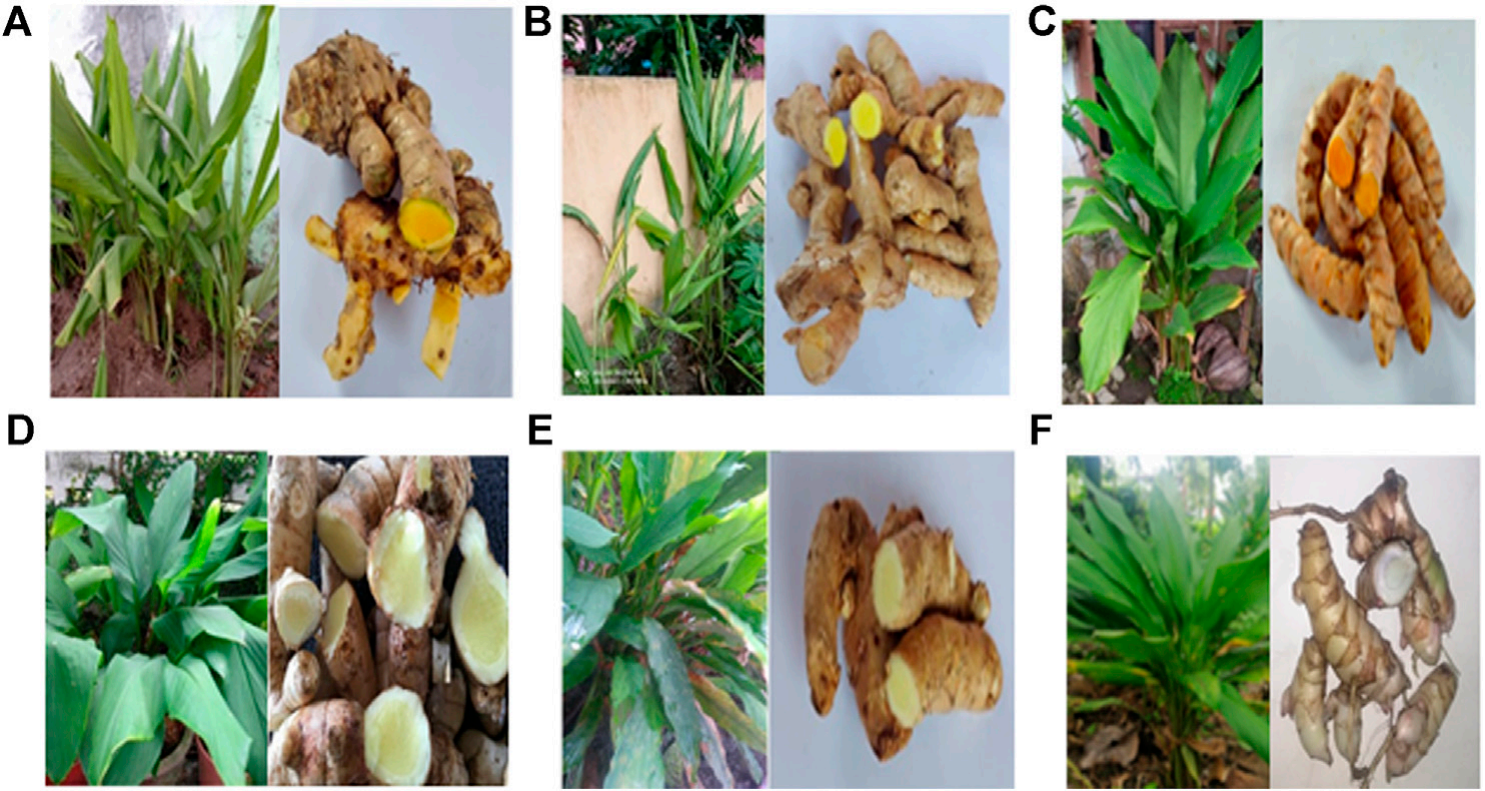
Plants and rhizomes of *Curcuma* species. **(A)** *Cucuma zanthorrhiza*, **(B)** *Curcuma mangga*, **(C)** *Curcuma longa*, **(D)** *Curcuma amada* (Artfire, 2016; Snapdeal, 2020), **(E)** *Curcuma zedoaria*, and **(F)** *Curcuma aeruginosa.*

*Curcuma* L*.* is one of the largest genera in the family of Zingiberaceae, and there are approximately 100 accepted *Curcuma* species. It is found throughout tropical Asia from India to South China, Southeast Asia, Papua New Guinea, and northern Australia (Dosoky and Setzer, 2018). The word “curcuma” is derived from the Arabic word “kurkum,” which means yellow color (Kaliyadasa and Samarasinghe, 2019). *Curcuma* species are originated from the Indo-Malayan region and widespread in Asia, Africa, and Australia (Sasikumar, 2005). Figure 1 shows the *Curcuma* species: *C. longa*, *C. zanthorrhiza*, *C. amada*, *C. mangga*, *C. aeruginosa*, and *C. zedoaria* that are discussed in this review. The rhizomes of these plants are widely utilized in traditional medicine and as spices, food flavors, colorants, cosmetics, and perfumery. *C. longa* Linn (syn. *Curcuma domestica* Val.) is native to tropical South Asia, but it has been found throughout tropical areas (Li et al., 2011), such as Cambodia, China, India, Nepal, Indonesia, Madagascar, Malaysia, the Philippines, and Vietnam (Yadav and Tarun, 2017). It is commonly called as turmeric (Li et al., 2011; HMPC, 2017; Rajkumari and Sanatombi, 2017) and the Golden Spice of India (Yadav and Tarun, 2017). *C. longa* has been associated to the Indian culture for nearly 4000 years and probably reached China by 700 AD, East Africa by 800 AD, and West Africa by 1200 AD (Yadav and Tarun, 2017). *C. longa* has a specific name in some regions, namely, Haridra (Sanskrit, Ayurvedic), Jianghuang (Chinese), Kyoo or Ukon (Japanese) (HMPC, 2017), kurkum (Arabic), and haldi (Hindi and Urdu) (Dosoky and Setzer, 2018). *C. longa* has yellow-white flowers, 10–15 cm of stalk length, the seeds are brown ovoid, the plant grows upright, and part used for spices and medicine is the rhizome (Tung et al., 2019). *C. zanthorrhiza* is native to Indonesia (Rajkumari and Sanatombi, 2017), and it has been established by the Food and Drug Supervisory Agency (BADAN POM) as one of the leading medicinal plants (Ervintari et al., 2019). It is known as temu lawak (Dewi et al., 2012) and Java turmeric (Kim M-B et al., 2014; Astana et al., 2018), and distributed in Southeast Asia. It has been grown in Thailand, the Philippines, Sri Lanka, and Malaysia (Oon et al., 2015). It is grown simply to produce rhizomes which are commonly used in folk medicine (Wahono et al., 2017b). It is an ethnomedicinal plant from Indonesia and Malaysia (Kim M-B et al., 2014). It has 2-m tall erect pseudostems (Rajkumari and Sanatombi, 2017) and is generally cultivated in village home gardens. The rhizomes smell balmy and taste bitter (Ilene et al., 2020). *C. zanthorrhiza* has been used as an active ingredient in cosmetic and hygienic products in Germany and the Netherlands (Drugbank, 2021).

*C. amada* is widely distributed in Myanmar, and in southern and eastern India. Apart from Myanmar, *C. amada* is also distributed in the tropics of Asia to Africa and Australia. It is widely cultivated in West Bengal, Gujarat, Tamil Nadu, and the northeastern states of India (Sasikumar, 2005). It has the resemblance with ginger (*Zingiber officinale*) but imparts a raw mango (*Mangifera indica*) flavor (Policegoudra et al., 2011). Thereby, it is usually known as mango ginger due to its mango flavor. The flavor has been attributed to the presence of *cis*-ocimene and car-3-ene (Ayodele et al., 2018). *C. amada* rhizomes are fleshy, buff colored, 5–10 cm long, and 2–5 cm in diameter (Artfire, 2016; Policegoudra et al., 2011; Snapdeal, 2020). *C. aeruginosa* is an endemic species in Myanmar, but it is also distributed in West Bengal and Kerala (Rajkumari and Sanatombi, 2017). *C. aeruginosa* is also an ethnomedicinal plant in Indonesia, Malaysia, Thailand, Northern Australia, and Papua New Guinea (Sulfianti et al., 2019). It is commonly known as Kali Haldi (in India) and has a deep-blue or bluish-black colored cortex with pungent odor. In Indonesia, *C. aeruginosa* is known as Temu Ireng (Choudhury et al., 2013; George and Britto, 2015), and in English, it is known as pink and blue ginger (Sulfianti et al., 2019), temu hitam in Malaysia, and waan-maha-mek in Thailand. It is a perennial herb derived from Burma and spread to tropical countries in Malaysia, Thailand, India, and Indonesia (Dosoky and Setzer, 2018). *C. zedoaria*, known as white turmeric, is a perennial herb with perpendicular pseudostem and fleshy roots. It is a native plant from Bangladesh, India, and Sri Lanka (Lobo et al., 2009), but it is a critically threatened species in Bangladesh and India (Anisuzzaman et al., 2008). It is known by several names in India, and the most common are Krachura (Sanskrit), Gandamatsi (Hindi), and Sutha (Bengali) (Lobo et al., 2009). In China, it is generally called Ezhu (Lee et al., 2019). *C. zedoaria* is widely cultivated in subtropical regions (Southeast Asia, Thailand, Indonesia, Japan, and China). From outside, *C. zedoaria* looks like ginger, but inside, it looks like turmeric (Dosoky and Setzer, 2018). *C. zedoaria* rhizome has dark orange-fleshed tubers (Rahayu et al., 2020). *C. mangga* rhizome is commonly known as mango turmeric as it has the mango-like smell as in *C. amada*. It is a perennial herb with 30–110 cm of stem height. It is native from Java (Rajkumari and Sanatombi, 2017). It is distributed in most tropical countries such as Indonesia, Thailand, and Malaysia (Hong et al., 2016).

## Ethnopharmacological Uses

*C. longa* is traditionally used as an antioxidant, anti-inflammatory, antidiabetic, hepatoprotective, and anticarcinogenic agent (Alsahli et al., 2018). It is well known as ethnomedicinal plant and used in different traditional systems in the world. In traditional medicine in Nigeria, *C. longa* is also used as an wound-healing agent (Adeshina et al., 2017). In Nepal, *C. longa* is applied as an anthelmintic, a tonic and blood purifier as well as for the treatment of Jaundice and liver disorder. In Peru, *C. longa* juice commonly known as Shapi natiyu is applied for the treatment of bronchitis and malaria. In Colombia, it is used for circulatory stimulant, healing wounds, liver cleaning, immune system booster, thrombosis, indigestion, diabetes, high cholesterol, and kidney infection (Ayati et al., 2019). The Ayurvedic Pharmacopoeia of India documented that *C. longa* is used as tonic, stomachic, and carminative. In Chinese Pharmacopoeia, *C. longa* has a potential for eliminating blood stasis, stimulating menstruation discharge, and relieving pain (Yue et al., 2010). In Pakistan traditional medicine, *C. longa* is used as a wound-healing agent and for the treatment of pimples. In Butanese folk medicine, it is known as Yung-ba and applied as tonic, antidote, antiseptic, anti-inflammatory, and as a good preservative (Ayati et al., 2019). *C. zanthorrhiza* is traditionally used for wound healing, as anti-inflammatory and anticarcinogecic agent, and for lowering of serum cholesterol levels (Kim et al., 2007) and booster immunity by Javanese (Setyati et al., 2019). In Malaysia, it is traditionally used to treat skin inflammation, rheumatism, stomach and liver disorders, and hepatitis (Kim M-B et al., 2014). In Ayurveda, *C. amada* is usually used for inflammation, asthma, bronchitis, biliousness, and skin disease (Policegoudra et al., 2006). The rhizomes are usually used for anorexia, dyspepsia, chronic ulcers, pruritus, gout, and inflammations (Thokchom and Phucho, 2015). Traditionally, *C. amada* is used for inflammation, stomach and skin diseases, cough, and rheumatism in Myanmar (Win et al., 2017). *C. aeruginosa* is used to booster immunity by Javanese (Setyati et al., 2019). It is used traditionally in Indonesia for gastrointestinal disease, and as antimicrobial and anti-inflammatory agents (Sulfianti et al., 2019). *C. zedoaria* is commonly known as white turmeric, and it is widely used as a traditional medicine in Indonesia (Putri, 2014; Aristyani et al., 2018), China and Japan (Kim et al., 2001), and India (Nan et al., 2014). *C. zedoaria* is traditionally used for treating cancer (Dutta, 2015) and also used as a traditional remedy to promote blood circulation in Korea and Japan (Kim et al., 2001). *C. zedoaria* is used to treat flatulent colic, hepatocirrhosis, and cancer in traditional Chinese medicine. It is also used to treat blood stagnation syndromes and for promoting menstruation (Carvalho et al., 2010). *C. mangga* is highly valued in Indonesian folk medicine for its healing properties in the treatment of stomach disorders, fever, and cancer-related diseases (Malek et al., 2011).

## Phytochemistry

Plants from the genus *Curcuma* L. have been intensively studied for their phytochemical contents and bioactivity due to their tremendous ethnopharmacological and therapeutic potentials. There are recent reviews on the phytochemistry, and biological and pharmacological activities of *Curcuma* species (Rajkumari and Sanatombi, 2017; Dosoki and Setzer, 2018; Chanda and Ramachandra, 2019; Kavitha and Mahadevi, 2020; USDA, 2021). Phytochemical analysis has revealed that *Curcuma* species are made up mainly of terpenoids, flavonoids, phenolic compounds, organic acids, anthocyanin, tannins, and inorganic compounds. Until now, phytochemical studies on 32 *Curcuma* species have isolated and identified a total of 719 compounds, which include 529 terpenoids, 15 flavonoids, 102 diphenylalkanoids, 19 phenylpropene derivatives, 3 alkaloids, 7 steroids, and 44 other types of compounds (Sun et al., 2017). The phytochemical content of *C. longa* has been extensively investigated, and more than 235 compounds have been identified in the rhizome, which are mainly polyphenols and terpenoids. The major group of polyphenols is curcuminoids, which may contain up to 80% of curcumin, and other two are demethoxycurcumin and bisdemethoxycurcumin. In total, there are 109 sesquiterpenes, 68 monoterpenes, 22 diarylheptanoids and diarylpentanoids, eight phenylpropene and other phenolic compounds, five diterpenes, four sterols, three triterpenoids, two alkaloids, and 14 other compounds (Li et al., 2011). The essential oils of flowers and leaves are mainly made up of monoterpenes, while the root and rhizome oils are dominated by sesquiterpenes. A recent study reported that the average essential oil content in the rhizome was 3.97%, and the major components identified by gas chromatography were ar-turmerone (40%), *α*-turmerone (10%), and curlone (23%) (Guimarães et al., 2020). Xanthorrhizol, a bisabolane-type sesquiterpenoid compound, is the major compound of *C. zanthorrhiza*. Curcumin, demethoxycurcumin, and bisdemethoxycurcumin are also present in appreciable amounts. Sesquiterpenes of the bisabolene-type and their oxygenated derivatives were reported to comprise more than 92% of the rhizome oil of *C. zanthorrhiza*. Xanthorrhizol (32%) was the most abundant sesquiterpene phenol. *ß*-Curcumene (17.1%), zingiberene (13.2%), *ß*-bisabolol (3.5%), and ar-curcumene (2.6%) were the other major components of the oil (Jantan et al., 2012).

Several valuable sesquiterpenoids such as zedoarondiol zedoalactone A, zedoalactone B, curcumenol, isocurcumenol, zedoarol, isofuranodiene, and furanodiene have been isolated from *C. aeruginosa* rhizome. The rhizome oil of this plant was made up mainly of 1, 8-cineol, *ß*-pinene, camphor, curzerenone, furanodienone, furangermenone, curcumenol, zedoarol, isocurcumenol, and *ß*-elemene (Jose and Thomas, 2014). *C. zedoaria* rhizome is rich in sesquiterpenoids which are represented by furanodienone, furanodiene, curzerenone, zedorone, germacrone, curzeone, 13-hydroxy germacrone, curcumenol, curcumenone, dihydrocurdione, zedoaronediol, dihydrocurdione, zedoarol, 13-hydroxygermacrone, curzeone curcumenone, curcumanolide-A, curcumanolide-B, *a*-turmerone, *ß*-turmerone, epicurzerenone, and curzerene. GC and GC-MS analyses of the rhizome oil revealed the presence of curzerenone (22.3%) as the major component, together with 1,8-cineole, germacrone, cymene, *a*-phellandrene, and *ß*-eudesmol (Lobo et al., 2009). Based on percent yield, myrcene (88.6%), ocimene (47.2%), and ar-turmerone (29.12%) were reported to be the major chemical constituents of *C. amada*. Other compounds that were present in appreciable amounts were (Z)-*β*-farnesene, guaia-6,9-diene, cis *ß*-ocimene, cis-hydroocimene, trans-hydroocimene, *a*-longipinene, *a*-guaiene, linalool, *ß*-curcumene, and turmerone (Jatoi et al., 2007).

The presence of these diverse bioactive compounds in the plants contributes to the diverse pharmacological activities. Curcumin, one of the main active ingredients in *Curcuma* species, has been widely reported for its strong immunomodulating, antioxidant, anti-inflammatory, and antitumor activities. Structure–activity relationship studies have revealed that the presence of different functional entities on the diarylheptanoid structure which include methoxy, phenoxy, and carbon–carbon double bonds was found to be responsible for the antioxidant property. However, the remarkable anti-inflammatory property was associated with the symmetry of the structure and position of substituents along with the number of methoxy groups. In addition, electron-withdrawing substituents and the α,β-unsaturated carbonyl group were indicated imperative for reactivity (Arshad et al., 2017). Besides the curcuminoids (curcumin, demethoxycurcumin, bisdemethoxycurcumin, and dihydrocurcumin), other compounds from *Curcuma* spp. with significant activity on the immune system include xanthorrhizol, turmeronol, curdione, curcuzedoalide, curcumenol, and germacrone.

## Immunomodulating Properties of *Curcuma* Species

*Curcuma* species and their bioactive compounds have been much investigated for their various biological and pharmacological activities, including antioxidant, anti-inflammatory, anticancer, hepato-protective, antifungal, antihypertensive, neuroprotective, and immunomodulatory effects through *in vitro* and *in vivo* studies. The six *Curcuma* species and their bioactive compounds discussed in this article have been documented to exhibit various pharmacological activities, particularly *via* modulation of the immune system. There are in-depth mechanistic studies on the immunomodulating effects of some of these species available in the literature. The immunomodulatory effects of the plant samples on the immune system are critically analyzed, and their underlying mechanisms of action are summarized in Table 1.

**TABLE 1:** Immunomodulatory activity of some *Curcuma* species.

Species	Subjects	Study design	Preparation	Immunomodulatory activities	Modulation	Parameters/mediators affected	References
*Curcuma amada* Roxb.	Rat PMNs	*in vitro*	Ethanol, petroleum ether, chloroform, and acetone extracts	Phagocytosis activity	↑	Phagocytosis	Karchuli and Pradhan (2011)
	Sheep RBC-induced albino Wistar rats	*in vivo*	Ethanol extract	Cellular immunity	↑	Delayed-type hypersensitivity response	Karchuli and Pradhan (2011)
	Sheep RBC induced-albino Wistar rats	*in vivo*	Ethanol extract	Humoral immunity	↑	Antibody titer	Karchuli and Pradhan (2011)
*Curcuma aeruginosa* Roxb.	Zymosan-stimulated human PMNs	*in vitro*	Methanol extract	ROS generation	↓	ROS	Jantan et al. (2011)
	Zymosan-stimulated macrophages of BALB/c mice	*in vitro*	Methanol extract	ROS generation	↓	ROS	Jantan et al. (2011)
	Human PMNs	*in vitro*	Methanol extract	PMN chemotaxis	↓	Chemotaxis	Jantan et al. (2011)
	Human whole blood	*in vitro*	Methanol extract	CD18/11a expression	↓	CD18/11a	Harun et al. (2015)
	Human whole blood	*in vitro*	Methanol extract	Phagocytosis activity	↓	Phagocytosis	Harun et al. (2015)
	Lymphocytes of BALB/c mice	*in vitro*	Extract by steam distillation	Counts of CD4^+^ and CD8^+^ cells	↑	CD4^+^ and CD8^+^ cells	Anggriani et al. (2019)
	DMBA-induced Wistar rats	*in vivo*	Ethanol extract	Cytokine release	↑	TNF-α, IFN-γ, IL-2, and IL-12	Sulfianti et al. (2019)
	*Epinephelus fuscoguttatus*	*in vivo*	*C. aeruginosa, Piper retrofractum*, and *C. zanthorrhiza* water extracts	Leukocyte number	↑	Total leukocyte count	Setyati et al. (2019)
	*Epinephelus fuscoguttatus*	*in vivo*	*C. aeruginosa, Piper retrofractum*, and *C. zanthorrhiza* water extracts	Phagocytosis activity	↑	Phagocytic index	Setyati et al. (2019)
*Curcuma longa* Linn	CMS-induced Sprague–Dawley rats	*in vivo*	Ethanol extract	Cytokine release	↓	IL-6 and TNF-α	Xia et al. (2006)
	Male Sprague–Dawley rats	*in vivo*	Ethanol extract	Splenic NK cell activity	↑	NK cell	Xia et al. (2006)
	Mice	*in vivo*	Methanol extract	Adaptive immune response	↑	Leukocytes number, antibody titer, spleen index, and delayed-type hypersensitivity response	Kumolosasi et al. (2018)
	Human peripheral blood mononuclear cells (PBMCs)	*in vitro*	Polar fraction of hot water extract	Proliferation response	↑	PBMC viability	Yue et al. (2010)
	Human peripheral blood mononuclear cells (PBMCs)	*in vitro*	Polysaccharide-enriched fraction at 200 μg/ml	Cytokine gene expression	↑	GM-CSF, IL-1, IL-5, IL-8, IL-10, and IL-13	Yue et al. (2010)
	Human peripheral blood mononuclear cells (PBMCs)	*in vitro*	Polysaccharide-enriched fraction at 400 and 800 μg/ml	Cytokine release	↑	TNF-α and IL-6	Yue et al. (2010)
	Human peripheral blood mononuclear cells (PBMCs)	*in vitro*	Polysaccharide-enriched fraction at 800 μg/ml	Cytokine release	↑	TGF-β	Yue et al. (2010)
	Human peripheral blood mononuclear cells (PBMCs)	*in vitro*	Polysaccharide-enriched fraction at 800 μg/ml	Lymphocyte population	↑	CD14^+^	Yue et al. (2010)
	Unstimulated mouse splenocytes and mouse macrophage (RAW264.7) cells	*in vitro*	Water extract	Cytokine release	↑	NO, IL-2, IL-6, IL-10, IL-12, IFN-γ, TNF-α, and MCP-1	Chinampudur et al. (2013)
	LPS stimulated mouse splenocytes	*in vitro*	Water extract	Cytokine release	↓	NO, IL-12, IL-6, and PGE_2_	Chinampudur et al. (2013)
	Con-A–induced splenocytes	*in vitro*	Water extract	Cytokine release	↑	IL-2 and IFN-γ	Chinampudur et al. (2013)
	Con-A–induced splenocytes	*in vitro*	Water extract	Cytokine release	↓	IL-10	Chinampudur et al. (2013)
	LPS-unstimulated and stimulated mouse splenocytes	*in vitro*	Polysaccharide fraction	Lymphocyte proliferation	↑	Splenocytes number	Chinampudur et al. (2013)
	LPS-stimulated mouse splenocytes	*in vitro*	Polysaccharide fraction	Cytokine release	↑	IL-10	Chinampudur et al. (2013)
	LPS-stimulated mouse splenocytes	*in vitro*	Polysaccharide fraction	Cytokine release	↓	IL-12 and PGE_2_	Chinampudur et al. (2013)
	RAW264.7 macrophages	*in vitro*	Water extract	Nitric oxide (NO) production	↑	NO levels	Pan et al. (2017)
	Diabetic infected rats	*in vivo*	Ethanol extract	Total IgE	↓	IgE levels	Shabana et al. (2020)
	Diabetic infected rats	*in vivo*	Ethanol extract	Leukocyte number	↓	Total leukocyte count (TLC)	Shabana et al. (2020)
	Diabetic infected rats	*in vivo*	Ethanol extract	NO production	↓	NO	Shabana et al., 2020
	Diabetic infected rats	*in vivo*	Ethanol extract	Cytokine release	↓	IL-6, TNF-a, and IL-1β	Shabana et al., 2020
	LP-BM5 MuLV-induced mice	*in vivo*	Alcohol extract	Proliferation	↓	T-cell, B-cell, and NK-cell	Kim O. K. et al. (2014)
	LP-BM5 MuLV-induced mice	*in vivo*	Alcohol extract	Cytokine imbalance	Prevented	Th1 (IL-2 and IFN-γ)/Th2) (IL-4 and IL-10)	Kim O. K. et al. (2014)
	C57BL/6 mice	*in vivo*	*C. longa*, Mulberry leaves, and purple sweet potato extracts	Proliferation	↓	T cell and B cell	Yoo et al. (2013)
	C57BL/6 mice	*in vivo*	*C. longa*, Mulberry leaves, and purple sweet potato extracts	Cytokine secretion	↓	Th 1 cytokines (IL-2 and IFN-γ), Th2 cytokines (TNF-α and IL-10)	Yoo et al. (2013)
	LP-BM5 MuLV-infected mice	*in vivo*	*C. longa* and sweet potato mixture	Messenger RNA (mRNA) expression	↑	MHC I and MHC II	Park et al. (2018)
	LP-BM5 MuLV-infected mice	*in vivo*	*C. longa* and sweet potato mixture	Population of CD4 (+)/CD8 (+) T cells	↓	CD4 (+)/CD8 (+) T cells	Park et al. (2018)
	LP-BM5 MuLV-infected mice	*in vivo*	C. longa powder and sweet potato mixture	Ig levels	↓	IgA, IgE, and IgG	Park et al. (2018)
	Human umbilical vein endothelial cells (HUVECs)	*in vitro*	Extract	mRNA levels	↓	NF-κB p65, IL-6, and TNF-α	Morales et al. (2012)
	C57BL mice	*in vivo*	Hot water extract	Cytokines release	↓	TNF-α, IL-6, and IL-6 m-RNA proteins	Uchio et al. (2017)
	*Fusarium root*	*in vivo*	Aqueous extract	mRNA of the defense-related genes	↑	Defensin and chitinase	Alsahli et al. (2018)
	*Clarias gariepinus*	*in vivo*	Powder	IgM level	↑	IgM	Adeshina et al. (2017)
	*Clarias gariepinus*	*in vivo*	Powder	Enzyme activity	↑	Lysozyme activity	Adeshina et al. (2017)
	*Cyprinus carpio*	*in vivo*	Powder	Leukocyte number	↑	Neutrophils, lymphocytes, monocyctes, eosinophils, and basophils	Arunkumar et al. (2016)
	Fish green terror (*Andinocara rivulatu*s)	*in vivo*	Powder	White blood cell number	↑	White blood cells	Mooraki et al. (2019)
	Nile tilapia (*Oreochromis niloticus*)	*in vivo*	Powder	Leukocrit levels	↑	Leukocrit number	Hassan et al. (2018)
	*M. rosenbergii*	*in vivo*	Powder	Gene expression	↑	Crustin and lysozyme	Alambra et al. (2012)
	Chicks	*in vivo*	Powder	Lymphocyte percentage	↑	Lymphocytes	Naderi et al. (2014)
*Curcuma zedoaria* Rosc.	LPS-stimulated RAW264.7 cells	*in vitro*	Methanol extract	NO production	↓	NO	Lee et al. (2019)
	LPS-stimulated RAW264.7 cells	*in vitro*	Methanol extract	Pro-inflammatory protein expression	↓	iNOS and COX-2	Lee et al. (2019)
	RBL-2H3 cells	*in vitro*	Aqueous acetone extract	Beta-hexosaminidase release	↓	Beta-hexosaminidase	Lobo et al. (2009)
	C57Bl/6J mice	*in vivo*	Ethanol extract	Total leukocytes count	↑	Leukocytes	Carvalho et al. (2010)
	*L. monocytogenes* and *S. aureus*–stimulated RAW264.7 cells	*in vitro*	Essential oil	Cytokine release	↓	TNF-α	Huang et al. (2019)
	PMA-stimulated RAW264.7 cells	*in vitro*	Polysaccharide fraction	Cytokine release	↑	TNF-α	Kim et al. (2001)
	PMA-stimulated RAW264.7 cells	*in vitro*	Polysaccharide fraction	NO production	↑	NO	Kim et al. (2001)
*Curcuma zanthorrhiza* Roxb.	Zymosan-stimulated human whole blood	*in vitro*	Methanol extract	ROS generation	↓	ROS	Jantan et al. (2011)
	Zymosan-stimulated PMNs	*in vitro*	Methanol extract	ROS generation	↓	ROS	Jantan et al. (2011)
	Zymosan-stimulated macrophages of BALB/c mice	*in vitro*	Methanol extract	ROS generation	↓	ROS	Jantan et al. (2011)
	Human PMNs	*in vitro*	Methanol extract	PMN chemotaxis	↓	Chemotaxis	Jantan et al. (2011)
	Human whole blood	*in vitro*	Methanol extract	Expression of CD18/11a	↓	CD18/11a	Harun et al. (2015)
	Human whole blood	*in vitro*	Methanol extract	Phagocytosis activity	↑	Phagocytosis	Harun et al. (2015)
	Hypercholesterolemic male Sprague–Dawley rats	*in vivo*	Curcuminoid cider	IL1β, TNFα, and chemokine gene expression	↓	IL1β, TNFα, and chemokine	Hardiwati et al. (2019)
	High cholesterol diet male Sprague–Dawley rats	*in vivo*	Curcuminoid cider	CD44, ICAM-1, iNOS, and LOX-1 gene expression	↓	CD44, ICAM-1, iNOS, and LOX-1	Mauren et al. (2016)
	Human lymphocytes	*in vitro*	Volatile oil	Lymphocytes proliferation	↑	Lymphocytes	Miksusanti (2012)
	Alcohol-induced mice	*in vivo*	Ethanol extract	Lymphocytes proliferation	↓	Lymphocytes	Ilene et al. (2020)
	High-fat diet-induced C57BL/6 mice	*in vivo*	Ethanol extract	Cytokine genes expression	↓	TNF-α, IL-6, IL-1β, and C-reactive protein (CRP)	Kim M-B et al. (2014)
	RAW 264.7 cells	*in vitro*	Crude polysaccharide extract	Chemical mediators release	↑	TNF-α and PGE_2_	Kim et al. (2007)
	RAW 264.7 cells	*in vitro*	Crude polysaccharide extract	Oxidative burst	↑	NO and H_2_O_2_	Kim et al. (2007)
	RAW 264.7 cells	*in vitro*	Crude polysaccharide extract	Phosphorylation	↑	IκBα	Kim et al. (2007)
	LPS-stimulated human gingival fibroblast-1 cells	*in vitro*	Crude polysaccharide extract	mRNA levels	↓	IL-1β, NF-κB p65, MMP-2, and MMP-8	Kim et al. (2018)
	HIV/AIDS patients	Clinical study	*C.* z*anthorrhiza* in combination with *C. mangga* and *Phyllanthus niruri*	Lymphocytes proliferation	Maintained	CD4^+^ value	Astana et al. (2018)
	Systemic lupus erythematosus (SLE) patients	Clinical study	*C. zanthorrhiza* supplementation with vitamin D3	Cytokine release	No significant difference reduction	IL-6	Wahono et al. (2017a)
	Systemic lupus erythematosus (SLE) patients	Clinical study	*C. zanthorrhiza* supplementation with vitamin D3	Cytokine release	No significant difference reduction	IL-17	Wahono et al. (2017b)
	Systemic lupus erythematosus (SLE) patients	Clinical study	Powder	Cytokine release	↓	TNF-α	Setiawati et al. (2017)
*Curcuma mangga* Val.	Swiss albino mice	*in vivo*	Ethanol extract and its fraction (hexane, chloroform, ethyl acetate, and aqueous fractions)	Paw and ear edema	↓	Paw and ear volume	Ruangsang et al. (2010)
	LPS and IFNγ–induced RAW264.7 macrophage cells	*in vitro*	Methanol extract	NO production	↓	NO	Abas et al. (2006)
	LPS-stimulated RAW264.7 macrophage cells	*in vitro*	Ethanol extract and chloroform, hexane, and ethyl acetate fractions	NO production	↓	NO	Kaewkroek et al. (2009)
	Zymosan-stimulated human whole blood	*in vitro*	Methanol extract	ROS inhibitory activity	↓	ROS	Jantan et al. (2011)
	Zymosan-stimulated human PMNs	*in vitro*	Methanol extract	ROS inhibitory activity	↓	ROS	Jantan et al. (2011)
	Zymosan-stimulated macrophages of BALB/c mice	*in vitro*	Methanol extract	ROS inhibitory activity	↓	ROS	Jantan et al. (2011)
	Human PMNs	*in vitro*	Methanol extract	PMN chemotaxis	↓	Chemotaxis	Jantan et al. (2011)
	Human whole blood	*in vitro*	Methanol extract	Expression of CD18/11a	↓	CD18/11a	Harun et al. (2015)
	Human whole blood	*in vitro*	Methanol extract	Phagocytosis activity	↑	Phagocytosis	Harun et al. (2015)
	Mice	*in vivo*	*n*-Hexane, ethyl acetate, and ethanol extracts	Phagocytosis activity	↑	Phagocytosis	Yuandani and Suwarso (2017b); Yuandani et al. (2019)
	Bovine RBC-stimulated mice	*in vivo*	Ethanol extract	Humoral immunity	↑	Antibody titer	Yuandani et al. (2018)
	Bovine RBC-stimulated mice	*in vivo*	Ethanol extract	Cellular immunity	↑	Delayed-type hypersensitivity response	Yuandani et al. (2018)
	Doxorubicin-induced immunosuppressive rats	*in vivo*	Ethanol extract	Humoral immunity	↑	Antibody titer	Yuandani et al. (2020)
	Doxorubicin-induced immunosuppressive rats	*in vivo*	Ethanol extract	Cellular immunity	↑	Delayed-type hypersensitivity response	Yuandani et al. (2020)

↑, increase.

↓, decrease.

### *Curcuma longa* L.

#### *In Vitro* Immunomodulating Effect of *C. longa*

Of all the *Curcuma* species investigated, the immunomodulatory effects of *C. longa* were the most studied. Interestingly, most experimental studies on the extracts of *C. longa* were carried out using *in vivo* animal models, and there were few *in vitro* studies. The followings are reports on the few *in vitro* studies that have been carried out to evaluate the immunomodulating effects of *C. longa*. *C longa* fermented by *Aspergillus oryzae* (FCL) exhibited immunomodulatory effects in RAW 264.7 cells. The different extracts of FLC on phagocytic activity, TNF-α, NO production, NK cell activity, and mRNA expression of LP-BM5 eco displayed the following results: hot water and 20% ethanol extracts increased the phagocytic activity, but there was no significant change in the production of NO relative to the control. There was also suppression of mRNA expression of LP-BM5 eco in FCL extracts and a four-fold increase in NK cell cytotoxity relative to the control group, especially in the 20% ethanol extract treatment group. However, TNF-α was significantly increased by the addition of FCL extracts (Yoo et al., 2014). Curcuminoid extract from *C. longa* has been reported to modulate TNF-⍺ and IL-6 at protein and gene levels in adipocytes *in vitro* (Hardiwati et al., 2019). *C. longa* decreased mRNA levels of NF-κB p65, IL-6, and TNF-α at 2.5–5 mg/L in LPS-induced human umbilical vein endothelial cells (HUVEC) (Morales et al., 2012). The polysaccharide extract isolated from *C. longa* was reported to possess immunostimulatory activities. Investigation of the polar fractions of *C. longa* hot water extract displayed that the extract stimulated PBMC proliferation using the [methyl-3H]-thymidine incorporation assay. Furthermore, its polysaccharide-enriched fraction at 200 μg/ml enhanced the cytokine expression (IL-1, IL-5, IL-8, IL-10, IL-13, and GM-CSF) detected by semiquantitatively using the antibody-based RayBio human cytokine array. However, the fraction at 200 μg/ml did not significantly enhance TNF-α, IFN-γ, TGF-β, and IL-6 productions. The production of IL-6 and TNF-α was only enhanced after treatment with the fraction at the higher doses of 400 and 800 μg/ml, respectively. The polysaccharide fraction at 800 μg/ml stimulated TGF-β release and CD14+ lymphocyte and population. However, the CD4+/CD8+ ratio was not altered after administration with polysaccharide fraction (Yue et al., 2010). In a related study, the immunostimulant and anti-inflammatory effects of *C. longa* aqueous extract and its polysaccharide fractions in the presence and absence of mitogens were determined. The extract enhanced splenocyte proliferation in unstimulated and LPS or concanavalin A-stimulated cells. The extract increased the levels of IL-2, IL-10, NO, IL-6, IL-12, TNF-α, IFN-γ, and MCP-1 in the absence of mitogen. Interestingly, *C. longa* extract decreased the levels of IL-12, IL-6, NO, and PGE-2 in LPS-stimulated cells, while TNF-α, IL-10, and MCP-1 levels were not altered. In contrast, the extract stimulated IL-2 and IFN-γ production but decreased IL-10 production from Con-A–induced splenocytes. Furthermore, its polysaccharide fraction showed stimulatory activity on lymphocyte proliferation in the absence or presence of LPS. The levels of IL-10 were increased, but the levels of IL-12 and PGE-2 were decreased after treatment with *C. longa* in LPS-stimulated cells (Chinampudur et al., 2013). In another study, a *C. longa* root aqueous extract standardized to a minimum of 20% of polysaccharides ukonan A, B, C, and D was shown to stimulate NO production in RAW264.7 macrophages (Pan et al., 2017).

#### *In Vivo* Immunomodulating Effect of *C. longa*

Most immunomodulating studies were carried out using aqueous and alcoholic extracts. The ethanol extract of *C. longa* was reported to suppress immune function, and behavioral and neuroendocrine alterations in a rat chronic mild stress (CMS) model. The enhancement of cytokine level (TNF-α and IL-6) activity and NK cell activity inhibition in the CMS-induced rat in splenocytes were reversed by administration of 35 mg/kg of *C. longa* ethanol extract and 7 mg/kg of fluoxetine as a control. The putative antidepressant properties of the extract were due to suppressive effects on cytokine biosynthesis. However, the extract increased the IL-6 level in the nonstress group, but there was no significant difference as compared with those of the normal group and caused a slight but no significant decrease in TNF-α levels. Although the extract enhanced splenic NK cell activity in CMS-treated rats, the NK cell activity of nonstressed rat did not change after treatment with *C. longa* (Xia et al., 2006). In another study, treatment with *C. longa* methanol extract with a single dose of 200 mg/kg for 14 days in mice stimulated innate and adaptive immunity. The effect of the extract on adaptive immunity was investigated by immunizing and challenging the mice with sheep red blood cells (sRBCs) on days 7 and 14, respectively. *C. longa* enhanced the adaptive immunity by increasing leukocyte number, antibody titer, spleen index, and delayed-type hypersensitivity response (Kumolosasi et al., 2018). However, the results of this study are preliminary as different doses of the extract need to be used to determine a dose–response relationship and the optimal dose for efficacy.

A previous study reported that treatment with *C. longa* in diabetic rats infected with *Staphylococcus aureus* resulted in a decrease of IgE, total leukocyte number (TLC), NO, and cytokine production (IL-6, IL-1β, and TNF-α). The results indicated that there was improvement of immune function by reducing levels of pro-inflammatory cytokines in the diabetic rats (Shabana et al., 2020). It was reported that 20% *C. longa* alcohol extract suppressed the increase of liver weights, lymph node, and spleen, and reduction of proliferation of T and B cells and NK cell activity stimulated by murine leukemia viruses–induced murine acquired immunodeficiency syndrome (AIDS) infection. Moreover, the extract suppressed Th1/Th2 (IL-2, IFN-γ/IL-4, and IL-10) cytokine imbalance and pro-inflammatory cytokine production (Kim O-K et al., 2014). This is in agreement with another study which showed that a diet consisted of *C. longa*; mulberry leaves and purple sweet potato extracts have the ability to prevent splenomegaly and lymphadenopathy induced by retrovirus, decrement of B- and T-cell proliferation, as well as reduction of Th 1 cytokine (IFN-γ and IL-2) release. It also reduced Th2 cytokine (TNF-α and IL-10) release (Yoo et al., 2013). Moreover, *C. longa* alone and in combination with purple sweet potato inhibited LP-BM5 murine leukemia virus (MuLV)-induced lymphadenopathy. The mixture of *C. longa* and purple sweet potato at the doses of 2 and 5 g/kg body weight increased the mRNA expression of MHC I and II as compared to those of the infected control group. The mixture at 5 g/kg body weight decreased the population of CD4^+^ T cells as compared to the infected control group, and also, the population of CD8^+^ T cells was lower than that of the normal group. Moreover, the extracts also affected T- and B-cell proliferation. The levels of Th1-type cytokines (IL-12 and IL-15) were enhanced after treatment by the mixture; meanwhile, Th2-type cytokine (IL-4, IL-10, IL-6, and TNF-α) production was significantly decreased as compared to the infected control group. In addition, the mixture at the doses of 2 and 5 g/kg decreased the levels of IgA, IgE, and IgG. Besides, *C. longa* alone or in mixture enhanced the phagocytosis activity of LP-BM5 MuLV-infected mice (Park et al., 2018). *C. longa* hot water extract protected the C57BL mice liver from acute injury induced by ethanol at 3 g/kg. The hepatic injury caused an increase in TNF-α, IL-6, and IL-6 m-RNA proteins. However, an increase in these proteins was not found in mice treated with hot water extract of *C. longa* 30 min before induction (Uchio et al., 2017). *C. longa* aqueous extract has been evaluated for its immunotherapeutic and hepatoprotective activities in CCl_4_ intoxicated Swiss albino mice. The aqueous extract reduced the levels of bilirubin and transaminase enzymes (SGOT and SGPT) in mice. Treatment with CCl_4_ resulted in liver damage and reduced nonspecific host–response parameters such as NO and MPO release, phagocytosis, intracellular killing capacity of peritoneal macrophages, and morphological alteration. Treatment with the extract also significantly protected the adverse effects of CCl_4_ on the nonspecific host response in the peritoneal macrophages of the mice (Sengupta et al., 2011).

Interestingly, there are several studies on the ability of *C. longa* to modulate the immune response of fish, chick, and prawn. *C. longa* increased plant defense by enhancing the defense-related genes such as defensin and chitinase of treated sunflower seedlings (Alsahli et al., 2018). The enhancement of host defense in fish has also been reported. *C. longa* leaf–enriched diet was fed to the fish to satiation twice daily for 12 weeks. Then, the fish was challenged with *Aeromonas hydrophila*. The highest stimulation on immunoglobulin M (IgM) level and lysozyme activity was observed in fish fed with 2.5% *C. longa*–fortified diets (Adeshina et al., 2017). A study reported that *Mesocyclops thermocyclopoides* enriched with *C. longa* enhanced the differential leukocyte number in fish (*Cyprinus carpio*), including enhancement of neutrophils, lymphocytes, monocytes, eosinophils, and basophils (Arunkumar et al., 2016). This result was supported by a previous study which reported the ability of 0.3% turmeric powder–enriched fish diet to enhance the white blood cell number significantly as compared to those of the control group (Mooraki et al., 2019). Turmeric in combination with rosemary (*Rosmarinus officinalis*) and thyme (*Thymus vulgaris*) increased the leukocrit levels in fish (Hassan et al., 2018).

*C. longa* was also able to enhance the immune response of prawns (*Macrobrachium rosenbergii*) after being infected by *Vibrio alginolyticus*. Identification using RT-PCR revealed that *C. longa*–enhanced feeds increased the gene expression of crustin and lysozyme in *M. rosenbergii*, indicating a remarkable increase in the expression of AMPs (antimicrobial peptides). Production of AMPs is a first-line host defense mechanism of innate immunity, and they are thought to be essential for organisms lacking adaptive immunity (Alambra et al., 2012). The ability of *C. longa* to modulate the immune response in chicks was also reported. *C. longa* powder constituted 2.5 and 7.5 g/kg of the diet, which significantly enhanced lymphocyte percentage in chicks. Supplementation of the diet with the powder at 2.5 g/kg of the diet resulted in a significant increase in anti-infectious bronchitis virus (IBV) titer compared to the control group (Naderi et al., 2014). In another study, 2.5% of *C. longa*–enriched diet protected chicken from *Salmonella pullorum* infection (Purwanti et al., 2018). Moreover, the cellular immunity of broiler chicken to phytohemagglutinin-P (PHA-P) was significantly higher in groups fed with higher amount of *C. longa*. The primary antibody titer to sRBCs was also stimulated (Sethy et al., 2017). These studies revealed that *C. longa* mostly enhanced the cellular and humoral responses of fish, chick, and prawns. Thus, this plant can be used as animal feed to enhance the immune defense of the animals.

Extensive cellular and animal studies have been performed to evaluate the immunomodulatory effects of *C. longa* by using various immune cells such as macrophages, monocytes, neutrophils, lymphocytes (T and B cells), and NK cells. There is a need to explore the immune effect of the plant with other immune cells, particularly the antigen-presenting cells such as dendritic cells. The existing reports should be supported by exploring the effects of the plant samples on various animal disease models of immune-related and chronic inflammatory disorders. All the extracts of *Curcuma* species used in the *in vitro* and *in vivo* immunomodulating studies were not analyzed for their chemical constituents or standardized to marker compounds. *C. longa* samples were mostly in the form of crude aqueous and alcoholic extracts. Some of the samples were curcuminoids or polysaccharide-rich extracts, but the chemical composition of the extracts were not determined. It has been suggested that the curcuminoids and polysaccharides might be the main contributors for immunomodulatory activity of the plant. The extracts used should be determined qualitatively and quantitatively by using validated analytical methods such as reversed-phase HPLC methods. Some of the bioactive compounds—especially the curcuminoids—have been isolated from the extracts, and their mechanistic effects in modulating the immune system have been determined.

### *Curcuma zanthorrhiza* Roxb.

#### *In Vitro* Immunomodulating Effect of *C. zanthorrhiza*

*C. zanthorrhiza* methanol extract has been reported to inhibit ROS generation in a luminol and lucigenin-enhanced chemiluminescence (CL) assay. *C. zanthorrhiza* rhizomes reduced ROS production from whole blood of human by *in vitro* study. Moreover, the extract significantly inhibited the release of ROS from zymosan-induced PMNs and macrophages. *C. zanthorrhiza* also showed strong inhibition on PMN migration, with an IC_50_ value of 2.5 μg/ml (Jantan et al., 2011). A previous study reported that the methanol extract of *C. zanthorrhiza* rhizomes showed strong inhibition on the expression of CD18/11a; meanwhile, the extract has low effect on leukocyte phagocytosis (Harun et al., 2015). The mRNA levels of IL-1β, NF-κB p65, MMP-2, and MMP-8 on LPS-induced human gingival fibroblast-1 cells were reduced after treatment with crude polysaccharide extract of *C. zanthorrhiza*. The extract of *C. zanthorrhiza* inhibited MAPK/activator protein-1 (AP-1) signaling pathways. *C. zanthorrhiza* has been documented to exhibit anti-inflammatory activities in LPS-induced RAW264.7 monocytes and H_2_O_2_-treated HT22 hippocampal cells (Kim et al., 2018).

#### *In Vivo* Immunomodulating Effect of *C. zanthorrhiza*

Curcuminoid cider, a traditional fermented product made by the addition of *Acetobacter xylinum* to curcuminoid fraction isolated from *C. zanthorrhiza*, reduced the gene expression of IL1β, TNFα, and chemokine in hypercholesterolemic rats (Hardiwati et al., 2019). The data were in accordance with a previous study which demonstrated the inhibitory activity of curcuminoid cider and curcuminoid fraction from *C. zanthorrhiza* on the gene expression of CD44, ICAM-1, iNOS, and LOX-1 in high-cholesterol diet rats (Mauren et al., 2016). Volatile oil from *C. zanthorrhiza* enhanced the lymphocyte proliferation from human male B blood type (Miksusanti, 2012). *C. zanthorrhiza* extract administration was able to reduce inflammatory lymphocytes in alcohol-induced hepatitis in mice (Ilene et al., 2020). *C. zanthorrhiza* ethanol extracts strongly reduced cytokine gene expression, which include TNF-α, IL-6, IL-1β, and C-reactive protein (CRP) in the liver, adipose tissue, and muscle of high-fat diet-induced obese mice (Kim M-B et al., 2014). The crude polysaccharide extract of *C. zanthorrhiza* consisted of glucose, galactose, arabinose, xylose, mannose, and rhamnose, and was also reported to significantly enhance the phagocytosis of macrophages and the production of NO, H_2_O_2_, TNF-α, and PGE_2_. In addition, it clearly enhanced phosphorylation of IκBα, suggesting a role as a NF-ĸB activator (Kim et al., 2007; Huang et al., 2010). *C. zanthorrhiza*–inhibited pro-inflammatory cytokine production in mice induced high-fat diet. *C. zanthorrhiza* extract at 100 mg/kg body weight/day decreased IL-1β gene expression by 89.9% compared to the control group (Ilene et al., 2020). *C. zanthorrhiza* was also reported to stimulate total and differential leukocytes in African catfish (*Clarias gariepinus*) (Lestari et al., 2019). *C. zanthorrhiza* rhizome in combination with *Zingiber officinale* rhizome, *Vitex trifolia* leaves, *Echinacea purpurea*, and citrus fruit in a herbal formula increased the number of macrophages phagocytizing *Candida albicans* as compared to those of *E. purpurea*–only group in mice. In addition, the herbal formula also displayed immunostimulatory activities on lymphocyte proliferation and the level of IgG actively phagocytizing *C. albicans* (Ikawati et al., 2019).

#### Clinical Studies of *C. zanthorrhiza* on the Immune System

An unsystematic clinical study of *C. zanthorrhiza* reported that *C. zanthorrhiza* extract reduced the population of B lymphocytes (Dewi et al., 2012). A previous study reported that *C. zanthorrhiza* in combination with *C. mangga* and *Phyllanthus niruri* maintained the levels of CD4^+^ in HIV/AIDS patients (Astana et al., 2018). *C. zanthorrhiza* in combination with *Vitex trifolia* did not cause liver and kidney damage after 14 days, 3 times a day treatment in women (Baroroh et al., 2011). Supplementation of *C. zanthorrhiza* with vitamin D3 was not able to decrease IL-6 level and elevate TGF-*β*1 systemic lupus erythematosus (SLE) in patients with hypovitaminosis D (Wahono et al., 2017b). These data were supported by a double-blind randomized controlled study on active SLE patients with hypovitaminosis D, which reported that addition of *C. zanthorrhiza* in vitamin D3 did not reduce IL-17 level as compared to those of singular vitamin D administration (Wahono et al., 2017a). Furthermore, a placebo-controlled double-blind clinical study showed that TNF-α release was reduced after treatment with the extract of *C. zanthorrhiza* for 4 weeks in SLE patients (Setiawati et al., 2017).

*C. zanthorrhiza* Roxb. is the second most popular plant among the genus *Curcuma* that has been investigated for its immunomodulating properties. Similar to *C. longa*, the crude extracts of *C. zanthorrhiza* were used in experimental studies to evaluate its *in vivo* immunomodulating effect using various animal models. There were a few *in vitro* studies, and the chemical constituents of the extracts were mostly not determined or the extracts were not standardized. Some clinical trials have been conducted on *C. zanthorrhiza* extracts, but they were unsystematic and not well designed. Despite the regulatory requirements for clinical studies and sufficient data not being generated on preclinical testing of *C. zanthorrhiza*, there were already reports on a few unsystematic case studies to evaluate the immunomodulating properties of *C. zanthorrhiza* in human. For clinical studies, sufficient preclinical testing should be generated using standardized extracts, which include bioavailability, and pharmacokinetic and toxicological studies, before they can be subjected to clinical studies.

### *Curcuma aeruginosa* Roxb.

#### *In Vitro* Immunomodulating of *C. aeruginosa*

The methanol extract of *C. aeruginosa* at 100 and 6.25 μg/ml showed moderate inhibition on CD18/11a expression on the surface of phagocytes, which was determined using a flow cytometry method. The extract at the same concentrations also demonstrated low inhibition on phagocytosis of leukocytes (Harun et al., 2015). Investigation on the effect of *C. aeruginosa* methanol extract on ROS generation from polymorphonuclear cells (PMNs) and peritoneal macrophages in human whole blood revealed that the extract possessed ROS inhibitory activity for luminol-stimulated chemiluminescence (CL). *C. aeruginosa* rhizomes inhibited oxidative burst of PMNs and macrophages, with IC_50_ values of 1.8 and 4.6 μg/ml, respectively. Interestingly, *C. aeruginosa* extract also possessed significant ROS inhibitory activity for lucigenin-enhanced CL. However, *C. aeruginosa* revealed low inhibition on PMN chemotaxis toward the chemoattractant, N-formyl-methionyl-leucyl-phenylalanine (fMLP), with percentage inhibition of 49.9% (Jantan et al., 2011).

#### *In Vivo* Immunomodulating of *C. aeruginosa*

*C. aeruginosa* extract, obtained by steam distillation, has been reported to increase the percentage of CD4^+^ and CD8^+^ cells (Anggriani et al., 2019). A previous study reported that *C. aeruginosa* ethanol extract was able to increase IFN-γ, TNF-α, IL-2, and IL-12 levels in 7,12-dimethylbenz [a]anthracene (DMBA)-induced Wistar rats. The highest stimulation on cytokines release was shown after treatment with the ethanol extract of *C. aeruginosa* at a dose of 80 mg/200 g body weight (Sulfianti et al., 2019). The aqueous extract of *C. aeruginosa* in combination with *Piper retrofractum* and *Curcuma zanthorrhiza* supplemented in a fish fed at the concentrations of 0.5, 1, and 1.5%, respectively, enhanced nonspecific immunity of *Epinephelus fuscoguttatus*. The addition of *C. aeruginosa* extract induced significant difference in the total leukocyte count of *Epinephelus fuscoguttatus* after being infected by *Vibrio alginolyticus* and *V. parahaemolyticus* during 15 days of observation. *C. aeruginosa* treatment increased the total leukocyte count on day 4 and day 8. Moreover, *C. aeruginosa* at concentration of 1% showed the strongest stimulation on phagocytosis activity, which was determined on day 8 (Setyati et al., 2019).

The *in vitro* and *in vivo* immunomodulating studies on *C. aeruginosa* were carried out on their crude aqueous and ethanol extracts. The bioactive metabolites contributing to the modulating effects were not identified. It is important to chemically characterize the extract to determine the bioactive compounds contributing to the immunomodulatory properties and mechanistic investigation to conclude the plant potency and effects on the immune-related disorders.

### *Curcuma zedoaria* (Christm.) Roscoe

#### *In Vitro* Immunomodulating Effect of *C. zedoaria*

*C. zedoaria* (Christm.) Roscoe rhizome extract has been reported to inhibit NO production from LPS-stimulated RAW264.7 cells. It has also been found to reduce iNOS and COX-2 expressions (Lee et al., 2019). In another study, *C. zedoaria* prevented *ß*-hexosaminidase release in RBL-2H3 cells and showed passive cutaneous anaphylaxis reaction in mice. *ß*-Hexosaminidase is a marker of antigen-IgE–mediated degranulation (Lobo et al., 2009). Essential oil from *C. zedoaria* was reported to reduce TNF-α release from *L. monocytogenes* and *S. aureus*–stimulated RAW264.7 cells (Huang et al., 2019). Polysaccharide fraction of *C. zedoaria* rhizome was found to enhance phagocytosis activity and splenocyte proliferation. It also stimulated the primary and secondary titers as well as delayed-type hypersensitivity response (Faradilla and Iwo, 2014). This work was supported by a previous study which showed that polysaccharide fraction of *C. zedoaria* enhanced phagocytosis of FITC-labeled Gram-negative bacteria (*E. coli*) or Gram-positive bacteria (*S. aureus*) by peritoneal macrophages. It also stimulated two microbicidal routes, oxygen-dependent and oxygen-independent mechanisms. Lysosomal activity increased after treatment with polysaccharide fraction as well as *in vivo* and *in vitro* respiratory burst. It was reported that PMA-induced respiratory burst of peritoneal macrophage was higher than those of RAW 264 cells identified using luminol-chemiluminescence–based assay. The production of H_2_O_2_, NO, and TNF-α was also enhanced at the doses of 10, 50, and 100 μg/ml, dose dependently (Kim et al., 2001).

#### *In Vivo* Immunomodulating Effect of *C. zedoaria*

The effect of *C. zedoaria* extract on tumor progression and peripheral blood cells in C57Bl/6J mice injected with B16F10 murine melanoma cells was determined using different routes of administration. A decrease in peritoneal cell number and a significant increase in total red and white blood cell counts were observed. Oral administration of the extract revealed a noteworthy increase only in the total leukocyte count (Carvalho et al., 2010). *C. zedoaria* has also been reported to stimulate immune response in fish. Supplemented diets with *C. zedoaria* increased the phagocytic rate and lysosome activity in *Epinephelus coioedes* fish. *C. zedoaria* was able to increase reactive oxygen production, identified using two different methods, NBT test and chemiluminescent assay (Nan et al., 2014).

Similar to the other *Curcuma* species already discussed, the metabolite profiles of *C. zedoaria* extracts were not determined. It is necessary to analyze the chemical constituents of the extracts or use standardized extracts in the studies as the phytochemical constituents of the plant may vary with variation in genetic adaptation of the plant population growing at different altitudes, its geographical distribution due to the changes in soil composition, and other environmental factors. Thus, using standardized extracts will ensure the dynamic change of varying amounts of phytochemical constituents in the plant is taken into consideration.

### *Curcuma mangga* Valeton & Zijp

#### *In Vitro* Immunomodulating Effect of *C. mangga*

A previous study reported *in vitro* NO inhibition activity of *C. mangga* which might contribute to its anti-inflammatory effect (Abas et al., 2006; Kaewkroek et al., 2009; Liu and Nair, 2011). Furthermore, *C. mangga* rhizome extract and its chloroform, hexane, and ethyl acetate fractions reduced NO production from LPS-induced RAW 264.7 cells. Among the fractions, the chloroform fraction showed the highest NO inhibition, followed by hexane, and then ethyl acetate fractions (Kaewkroek et al., 2009). A previous study of the methanol extract of *C. mangga* rhizomes on whole blood showed that the extract exhibited strong inhibitory activity upon activation by zymosan. *C. mangga* rhizome extract possessed high ROS inhibitory activity in PMNs and peritoneal macrophages as investigated in a luminol-enhanced CL assay. The extract also inhibited the release of ROS from PMNs and macrophages in a lucigenin-enhanced CL assay, with IC_50_ values of 0.9 and 6.6 μg/ml, respectively (Jantan et al., 2011). *C. mangga* methanol extract has also been found to significantly suppress the cell surface expression of CD18/11a as compared to the negative control. However, the extract of *C. mangga* rhizome at the concentration of 100 and 6.25 μg/ml showed immunostimulatory activity on phagocytosis of leukocytes (Harun et al., 2015).

#### *In Vivo* Immunomodulating Effect of *C. mangga*

*C. mangga* Valeton & Zijp rhizome ethanol extract, its different organic fractions (hexane, chloroform, and ethyl acetate), and aqueous fraction have showed appreciable anti-inflammatory and analgesic activities in mice and inflammatory models using croton oil-induced mouse ear edema and carrageenan-induced rat paw edema. The plant extract and its fractions at 200 mg/kg demonstrated analgesic activity by reducing the number of writhing and also produced antinociception using hot plate and formalin test. At 200 mg/kg, the hexane and chloroform fractions significantly prolonged the latency time, but ethyl acetate and aqueous fractions were not active. In addition, the ethanol extract of *C. mangga* rhizome and its fractions displayed significant reduction of paw and ear edema in rat (Ruangsang et al., 2010). Our previous study reported that the n-hexane, ethyl acetate, and ethanol extracts of *C. mangga* rhizomes at the doses of 100, 200, and 400 mg/kg increased the carbon clearance rate, indicating the enhancement of carbon engulfment by cells in the reticuloendothelial system of mice, thus stimulating the phagocytosis activity in mice (Yuandani and Suwarso, 2017a; Yuandani et al., 2019). In addition, the *C. mangga* rhizome ethanol extract exhibited stimulation of antibody titer against bovine red blood cells in a dose-dependent way by using the hemagglutination method. The cellular immunity was also enhanced after treatment with *C. mangga* ethanol extract by increasing the bovine red blood cell–induced mice paw volume (Yuandani et al., 2018). Moreover, the ethanol extract of *C. mangga* rhizome stimulated the immune response in doxorubicin-induced immunosuppressive rats, which was indicated by the elevation of antibody titer and delayed hypersensitivity (DTH) response (Yuandani et al., 2020).

As with other *Curcuma* species already discussed, the effects of *C. mangga* on the immune cells and experimental animals may vary considerably, depending on the experimental conditions used, including the solvent of extraction, extraction method, cell line, animal model, treatment scheme, and different disease animal models. Dosage and concentration of raw extracts of the plant are crucial in order to achieve the desired benefit. Thus, to ensure the results are reproducible when the study is replicated, the same methodology has to be used by other researchers.

### *Curcuma amada* Roxb.

The ethanol, petroleum ether, chloroform, and acetone extracts of *C. amada* enhanced the phagocytosis activity of PMNs. The ethanol extract at a concentration 3 mg/ml showed the highest stimulation on percentage of phagocytosis. Further study on delayed hypersensitivity response against sRBCs showed that the ethanol extract of *C. amada* increased the paw volume. Moreover, the ethanol extract at the doses of 100, 200, and 400 mg/kg enhanced the antibody titer dose-dependently (Karchuli and Pradhan, 2011). Supercritical carbon dioxide (CO_2_) extract prepared from *C. amada* rhizomes has potential to be used for the treatment of immune disorder such as autoimmune diseases. Specifically, the extract can be used to treat or prevent hypersensitivity diseases, in particular IgE-mediated allergic reactions as well as autoimmune disorders (Weidner et al., 2001). *C. amada* in combination with *Tinospora cordifolia*, *Piper longum*, and *Albizia lebbeck* in a herbal preparation can be used to treat allergy (Palpu et al., 2008). The chemical constituents responsible for eliciting the activity were not determined, although a few potent activities have been reported on *C. amada* extract. There is a need to proceed to study in detail the underlying mechanisms on relevant signaling events followed by *in vivo* studies to explore the potential of this plant as a natural immunomodulating agent.

### Immunomodulatory Effects of Bioactive Compounds of *Curcuma* Species

**FIGURE 2:**
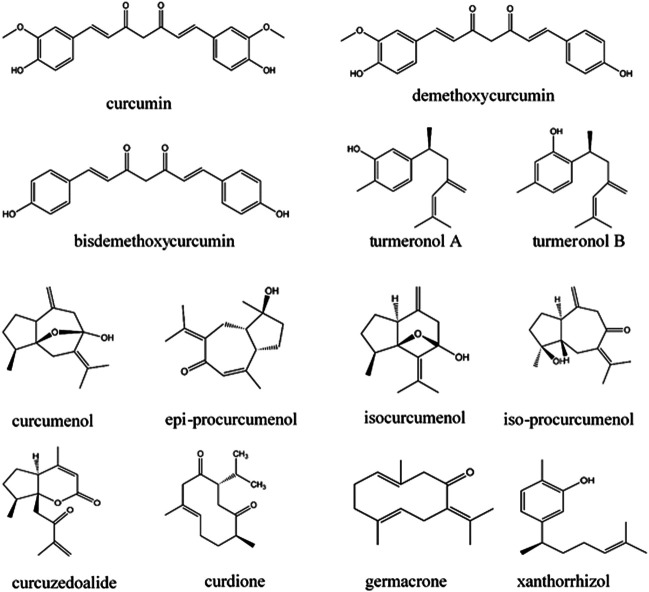
Chemical structures of potential immunomodulators from *Curcuma* species.

Plants in the genus *Curcuma* contain many compounds which contribute to the immunomodulatory activity of the plants, as shown in Table 2. Among the compounds from *Curcuma* species, curcumin and xanthorrhizol have been discussed in detail in this review as they have been widely investigated for their immunomodulating effects on the innate and adaptive immune system. Other compounds including turmeronols, curdione, curcuzedoalide, demethoxycurcumin, bisdemethoxycurcumin, dihydrocurcumin, curcumenol, epi-procurcumenol, isocurcumenol, and iso-procurcumenol germacrone are also included in this review, but their data are limited as they have not been well investigated for their immunomodulating effects. The chemical structures of these compounds are included in Figure 2.

**TABLE 2:** Bioactive compounds of *Curcuma* species with immunomodulating activity and their mechanisms of action.

Main compound	Species	Subjects	Study design	Immunomodulatory activities	Modulation	Parameters/mediators affected	References
Curcumin	*Curcuma* species	High glucose-cultured monocytes	*in vitro*	Cytokine production	↓	IL6, IL8, TNFα, and MCP1	Jain et al. (2009)
		Streptozotocin-induced rats	*in vivo*	Cytokine production	↓	IL6, TNFα, and MCP1	Jain et al. (2009)
		Mice pancreatic	*in vivo*	Leukocyte infiltration	↓	Leukocytes	Castro et al. (2014)
		M-stimulated BDC2.5-splenocytes	*in vitro*	T-cell proliferation	↓	CD4^+^, T cells, and IFN-γ	Castro et al. (2014)
		BDC2.5 mice T lymphocite	*in vitro*	T-cell proliferation	↓	T lymphocyte	Castro et al. (2014)
		PMN leukocytes	*in vitro*	DHA synthesis	↑	DHA	Pisani et al. (2009), Wu et al. (2015)
		PMN leukocytes	*in vitro*	ROS production	↓	ROS	Pisani et al. (2009), Wu et al. (2015)
		LPS-induced mice mastitis	*in vivo*	Myeloperoxidase activity	↓	MPO	Fu et al. (2014)
		LPS-induced mice mastitis	*in vivo*	Cytokine production	↓	TNF-α, IL-6, IL-1β, and TLR4	Fu et al. (2014)
		LPS-induced mice mastitis	*in vivo*	Phosphorylation	↓	IκB-α and NF-κB p65	Fu et al. (2014)
		Microglial cells	*in vitro*	NO production	↓	NO	Cianciulli et al. (2016)
		Microglial cells	*in vitro*	Phosphorylation	↓	IL-1β, IL-6, TNF-α, and PI3K/Akt	Cianciulli et al. (2016)
		Microglial cells	*in vitro*	NF-κB and iNOS expression	↓	NF-κB and iNOS	Cianciulli et al. (2016)
		Microglial cells	*in vitro*	Cytokine production	↓	NO, PGE_2_, TNF-α, iNOS, and COX-2	Yu et al. (2018)
	*C. longa*	Healthy albino mice	*in vivo*	White blood cells production and weight lymphoid	↑	Lymphoid organs and white blood cells	Afolayan et al. (2018)
		Dendritic cells	*in vitro*	Surface molecule expression	↓	CD80, CD86, MHC class II, and IL-1	Kim et al. (2005)
		Dendritic cells	*in vitro*	Cytokine production	↓	IL-6, IL-12, and TNF- α	Kim et al. (2005)
		Dendritic cells	*in vitro*	NF-κB p65 translocation	↓	NF-κB p65	Kim et al., 2005
		Bronchoalveolar of Balb/c mice	*in vivo*	Allergic response	↓	Eosinophils	Ravikumar and Kavitha (2020)
		Bronchoalveolar of Balb/c mice	*in vivo*	Cytokine production	↓	IL-4	Ravikumar and Kavitha (2020)
		PBMCs	*in vitro*	T-cell proliferation	↓	Lymphocyte	Yadav et al. (2005)
		PBMCs	*in vitro*	Cytokine production	↓	IL-2 and TNF-α	Yadav et al. (2005)
		PBMCs	*in vitro*	NF-κB	↓	NF-κB	Yadav et al. (2005)
		Erythroleukemic cell line K562	*in vitro*	Cytotoxicity	↑	NK cell	Yadav et al. (2005)
		Lupus BALB/c mice	*in vivo*	Adaptive immune response	↓	Th1, Th2, and Th17	Kalim et al. (2017)
		Lupus BALB/c mice	*in vivo*	ANA levels	↓	ANA	Kalim et al. (2017)
		Monocytes and liver macrophages	*in vivo*	ROS production	↓	ROS	Inzaugarat et al. (2017)
		Monocytes	*in vivo*	TNF-α and IFN- γ production	↑	TNF-α and IFN- γ	Inzaugarat et al. (2017)
	*C. longa*	Fish	*in vivo*	Immune response	↑	Immune	Alambra et al. (2012)
	*C. zedoaria*	RBL-2H3 cells	*in vitro*	beta-Hexosaminidase production	↓	Beta-hexosaminidase	Matsuda et al. (2004)
		RBL-2H3 cells	*in vitro*	Cytokine production	↑	TNF–α and IL–4	Matsuda et al. (2004)
Turmeronol	*C. longa*	RAW264.7 cells	*in vitro*	PGE_2_ and NO production	↓	PGE_2_ and NO	Okuda-Hanafusa et al. (2019)
		RAW264.7 cells	*in vitro*	Cytokine production	↓	IL-1β and IL-6	Okuda-Hanafusa et al. (2019)
		Cytoplasm into the nucleus	*in vitro*	NF-κB translocation	↓	NF-κB	Okuda-Hanafusa et al. (2019)
Curdione	*C. aeruginosa*	CD95 protein	*in silico*	Docking score	↓	Curdione to CD95	Anggriani et al. (2019)
1,8-cineol		CD95 protein	*in silico*	Docking score	↑	1,8-cineol to CD95	Anggriani et al. (2019)
Isocurcumenol		Chicken embryo fibroblast	*in vitro*	Toxicity	-	Fibroblast cells and lymphocytes	Lakshmi et al. (2011)
Isoprocurcumenol		RAW264.7 cells	*in vitro*	NO activity	↓	NO	Lee et al. (2019)
Germacrone	*C. zedoaria*	RAW264.7 cells	*in vitro*	NO activity	↓	NO	Lee et al. (2019)
Curzerenone		RAW264.7 cells	*in vitro*	NO activity	↓	NO	Lee et al., 2019
Curcumenol		RAW264.7 cells	*in vitro*	NO activity	↓	NO	Lee et al. (2019)
Curcuzedoalide		RAW264.7 cells	*in vitro*	NO activity	↓	NO	Lee et al. (2019)
		RAW264.7 cells	*in vitro*	iNOS and COX-2 response	↓	iNOS and COX-2	Lee et al. (2019)
Dihydrocurcumin		RBL-2H3 cells	*in vitro*	beta-Hexosaminidase production	↓	beta-Hexosaminidase	
		RBL-2H3 cells	*in vitro*	Cytokine production	↑	TNF-α and IL-4	Matsuda et al. (2004)
Tetrahydrodemethoxycurcumin		RBL-2H3 cells	*in vitro*	beta-Hexosaminidase production	↓	beta-Hexosaminidase	Matsuda et al. (2004)
		RBL-2H3 cells	*in vitro*	Cytokine production	↑	TNF-α and IL-4	Matsuda et al. (2004)
Tetrahydrobisdemethoxycurcumin		RBL-2H3 cells	*in vitro*	Hexosaminidase production	↓	beta-Hexosaminidase	Matsuda et al. (2004)
		RBL-2H3 cells	*in vitro*	Cytokine production	↑	TNF-α and IL-4	Matsuda et al. (2004)
1,7-bis(4-hydroxyphenyl)-1,4,6-heptatrien-3-one		Lipopolysaccharide (LPS)-activated macrophages	*in vitro*	TNF-α production	↓	TNF-α	Jang et al. (2001)
		Macrophages	*in vitro*	NO production and iNOS expression	↓	NO and iNOS	Jang et al. (2004)
Procurcumenol		lipopolysaccharide (LPS)-activated macrophages	*in vitro*	TNF-α production	↓	TNF-α	Jang et al. (2001)
Xanthorrhizol	*C. zanthorrhiza*	Human gingival fibroblast-1 cells	*in vitro*	mRNA levels	↓	IL-1β, NF-κB p65, MMP-2, and MMP-8	Kim et al. (2018)
		RAW 264.7 cell line	*in vitro*	MAPK and AP-1 response	↓	MAPK and AP-1	Kim et al. (2018)
Demethoxycurcumin	*C. mangga*	RAW 264.7 cell line	*in vitro*	NO production	↓	NO	Kaewkroek et al. (2009)
		RAW 264.7 cell line	*in vitro*	NO and PGE_2_ production	↓	NO and PGE_2_	Kaewkroek et al. (2010)
		RAW 264.7 cell line	*in vitro*	mRNA expressions	↓	iNOS and COX-2	Kaewkroek et al. (2010)
Bisdemethoxycurcumin		RAW 264.7 cell line	*in vitro*	NO production	↓	NO	Kaewkroek et al. (2009)
		RAW 264.7 cell line	*in vitro*	NO and PGE_2_ production	↓	NO and PGE_2_	Kaewkroek et al. (2010)
		RAW 264.7 cell line	*in vitro*	mRNA expressions	↓	iNOS and COX-2	Kaewkroek et al. (2010)
4-[(1R, 4aR, 8aR)-decahydro-5, 5, 8a-trimethyl-2-methylene-1-naphthalenyl]-, (3E)-rel		RAW 264.7 cell line	*in vitro*	NO and PGE_2_ production	↓	NO and PGE_2_	Kaewkroek et al. (2009)
		RAW 264.7 cell line	*in vitro*	mRNA expressions	↓	iNOS and COX-2	Kaewkroek et al. (2009)
15,16 bisnorlabda-8(17), 11-dien-13-one		RAW 264.7 cell line	*in vitro*	NO and PGE_2_ production	↓	NO and PGE_2_	Kaewkroek et al. (2010)
		RAW 264.7 cell line	*in vitro*	mRNA expressions	↓	iNOS and COX-2	Kaewkroek et al. (2010)
(E)-15,15-diethoxylabda-8 (17),12-dien-16-al		RAW 264.7 cell line	*in vitro*	NO and PGE_2_ production	↓	NO and PGE_2_	Kaewkroek et al. (2010)
		RAW 264.7 cell line	*in vitro*	mRNA expressions	↓	iNOS and COX-2	Kaewkroek et al. (2010)

↑, increase.

↓, decrease.

-, no changes.

### Curcumin

It is a major compound of *C. longa* and can also be found in other *Curcuma* species. This natural diarylheptanoid compound has been mainly isolated from the rhizomes of *C. longa* and studied extensively for various pharmacological activities, including antioxidant, anti-inflammatory, immunomodulatory, antiangiogenic, anticancer, antiproliferative, and proapoptotic. It has been one of the most intensively investigated compounds for its immunomodulatory properties. Many preclinical investigations which include *in vitro* cell assays and *in vivo* studies in animal models have been carried out on curcumin to evaluate its modulatory effects in the immune system. It is also undergoing extensive clinical trials based on its anti-inflammatory properties for the treatment of cancer.

#### *In Vitro* Immunomodulating Effect of Curcumin

**FIGURE 3:**
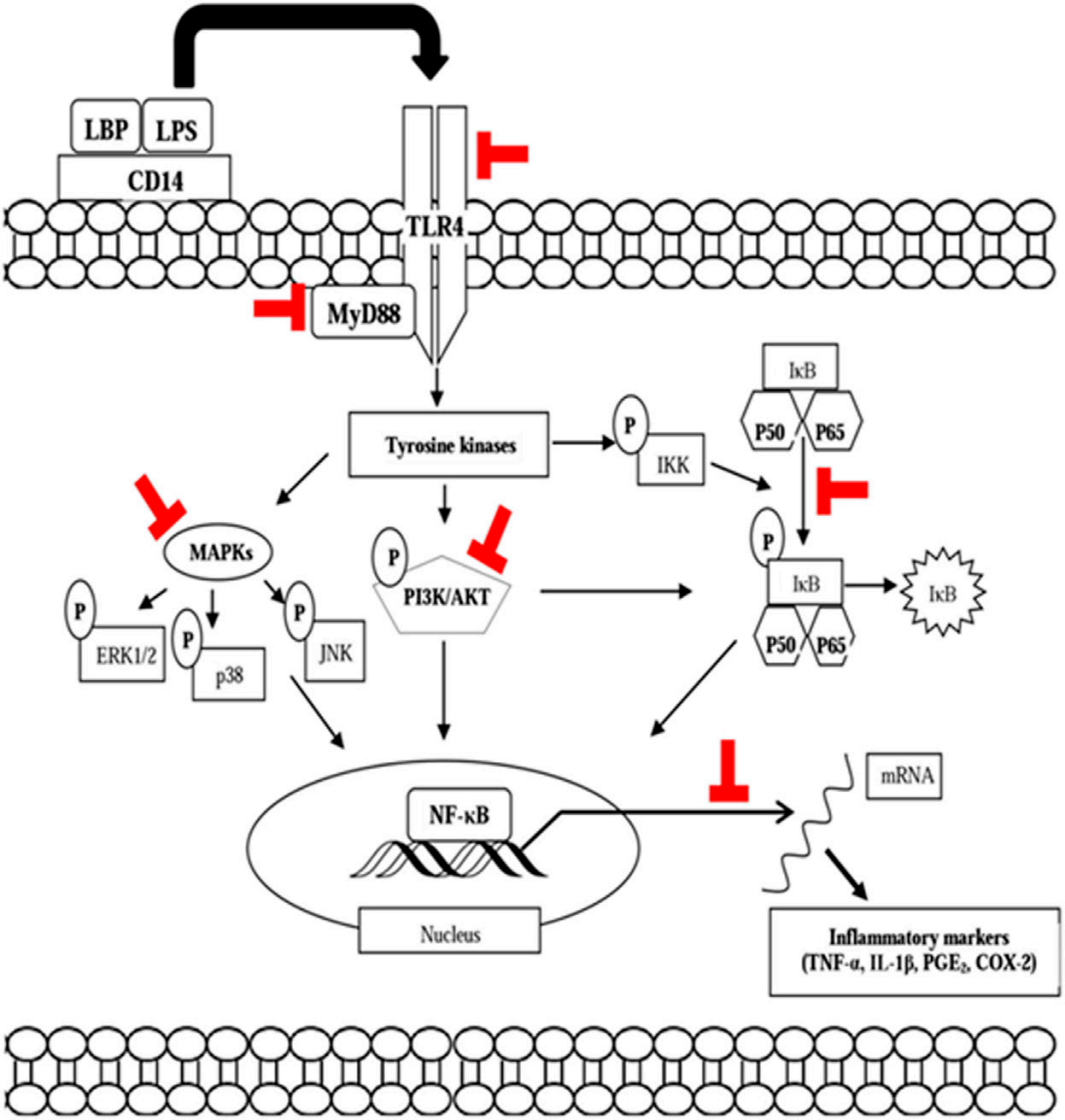
Modulatory effects of curcumin on the NF-κB, MAPK, and Akt signaling pathways. The thick red block sign indicates the possible point of modulation of the signal transduction pathways. NF-κB, nuclear factor kappa β; MAPK, mitogen-activated protein kinase; PI3K/Akt, phosphatidylinositol 3-kinase and protein kinase B; P, phosphoryl group.

The immunomodulating activity of curcumin has been demonstrated by many *in vitro* studies using several immune cells. Curcumin has been shown to inhibit inflammatory responses by suppressing COX-2 and NO, NF-ĸB, iNOS, and lipoxygenase in IFN-γ or TNF-α–activated macrophages and NK cells (Surh et al., 2001). A study by Jain et al. (2009) revealed that curcumin significantly reduced the production of IL-6, IL-8, TNF-α, and MCP-1 from high glucose-cultured monocytes. Low concentration of curcumin reduced NOS activity and NO production from macrophages. In another study, curcumin inhibited the immunostimulatory function of dendritic cells, leading to the reduction of CD80, CD86, and MHC class II expression, but not MHC class I expression as well as IL-12 expression and cytokine release (IL-1, IL-6, and TNF- α). Curcumin also inhibited LPS-induced MAPK activation and the translocation of NF-B p65 as well as impaired induction of Th1 responses (Kim et al., 2005). Gao et al. (2004) demonstrated that curcumin inhibited pro-inflammatory cytokine (TNF-α, IL-1, IL-12, and IL-6) expression in PMA or LPS-activated macrophages, dendritic cells, monocytes, and splenic lymphocytes. Curcumin has also been found to suppress PHA-stimulated lymphocyte proliferation and IL-2 release as well as transcription factor NF-KB and TNF-α production from LPS-stimulated PBMC to enhance NK cell cytotoxicity (Yadav et al., 2005). Curcumin has been shown to be able to increase ω-3 polyunsaturated fatty acid (PUFA) synthesis in the brain. An *in vitro* study has showed that PUFAs have beneficial effects on stimulating immune response by stimulating neutrophil phagocytosis activity, while decreasing the release of ROS in goat neutrophils (Pisani et al., 2009; Wu et al., 2015). Curcumin also increased ROS production from linoleic acid–stimulated monocytes and liver macrophages as well as TNF-α production from leptin-induced monocytes and IFN-γ release from CD4^+^ T cells (Inzaugarat et al., 2017). Pretreatment with curcumin significantly reduced the production of NO, the expression and release of TNF-α, IL-1β, IL-6, PI3K/Akt phosphorylation, NF-κB activation, and iNOS expression in LPS-stimulated microglial cells (Cianciulli et al., 2016). In addition, NO, PGE_2_, and TNF-α production as well as iNOS and COX-2 expression in LTA-activated microglial cells were reduced by curcumin (Yu et al., 2018). In an *in silico* study, curcumin has been shown to bind to viral S1 protein, which is important for SARS-CoV-2 entry; hence, it may prevent cytokine storm in a severe form of COVID-19 (Pawitan, 2020). The pathways and inflammatory mediators involved in the immunomodulating property of curcumin are illustrated in Figure 3.

#### *In Vivo* Immunomodulating Effect of Curcumin

*In vivo* studies to determine the immunomodulatory effects of curcumin were carried out using several animal models. Curcumin reduced the levels of IL6, TNFα, and MCP1 in streptozotocin-induced type 1 diabetes rats. Moreover, curcumin prevented pancreatic leukocytes infiltration that might initiate *ß*-cell destruction. In addition, curcumin reduced CD4^+^ T cell proliferation and IFN-γ release from M-stimulated BDC2·5-splenocytes as well as reduced LPS/IFN-γ–induced dendritic cell maturation. Antigen-specific T-lymphocyte proliferation has also been reduced by curcumin action on both T cells and antigen-presenting cells (APCs) (Castro et al., 2014). Administration of curcumin to lactating mice prevented mice mastitis by reducing the MPO activity; expression of TNF-α, IL-6, IL-1β, TLR4; and phosphorylation of IκB-α and NF-κB p65 after being induced by LPS (Fu et al., 2014). Nanoparticulate curcumin demonstrated stronger activity on cellular and humoral immunity, and increased lymphoid organs and white blood cell production than those of control (Afolayan et al., 2018). A previous study reported that curcumin displayed immunomodulatory effects in comorbid diabetic asthma mice by reducing eosinophil number and Il-4 level with a high IFN-γ to IL-4 ratio in the blood and bronchoalveolar after ovalbumin injection (Ravikumar and Kavitha, 2020). Administration of curcumin to pristane-induced lupus mice decreased Th1, Th2, and Th17 and slightly increased Treg percentages. In addition, ANA levels were also decreased after curcumin treatment (Kalim et al., 2017). Curcumin was able to enhance immune response in *Macrobrachium rosenbergii* after being challenged with *Vibrio alginolyticus* (Alambra et al., 2012).

#### Clinical Studies of Curcumin on Immune System

Presently, curcumin is under extensive clinical investigation where there are 116 ongoing clinical trials on curcumin, the status of which can be found on http://www.clinicaltrials.gov/. Among the clinical trials on curcumin, 99 of them were based on its anti-inflammatory properties. Cancer (e.g., breast, pancreatic, lung, colorectal, and prostate), inflammatory bowel diseases (IBD; Crohn’s disease and ulcerative colitis) and rheumatoid arthritis were the major diseases for which trials had been conducted, reflecting the pleiotropic actions of curcumin. In these trials, curcumin often acted as a dietary supplement or an adjunct treatment to the standard therapy. Studies on the efficacy and safety of curcumin as adjuvant in the treatment of cancer and cognitive damage will continue to dominate in future clinical trials (Jurenka, 2009). In their review on the clinical effects of curcumin in ulcerative colitis, Kumar et al. (2012) suggested that curcumin may be a safe and effective therapy for the maintenance of remission when given as adjunct therapy in quiescent ulcerative colitis. However, the results were preliminary due to the low number of enrolled patients participated in the clinical trials, as suggested by Fürst and Zündorf (2014). They suggested more thorough controlled randomized trials are required to be pursued to determine the safety level and efficacy of the compound for human use. The success of curcumin as a potent anti-inflammatory agent in future depends on the findings of high-quality and big cohort studies. Moreover, curcumin has poor bioavailability, and many studies have been carried out to address this issue *via* chemical and technological methods. Preparation of more stable curcumin derivatives and use of nanotechnology for curcumin delivery are actively being pursued to improve the bioavailability of curcumin (Anand et al., 2007). Curcumin still has potential to be used clinically for the treatment of the abovementioned indications as it is nontoxic with good safety profile and well tolerated.

### Xanthorrhizol

Xanthorrhizol is a bisabolane-type sesquiterpenoid, isolated from *C. zanthorrhiza* Roxb. It is known to possess diverse pharmacological activities, including antioxidant, anti-inflammatory, antimicrobial, anticancer, hepatoprotective, nephroprotective, antihypertensive, antihyperglycemic, antiestrogenic, and antiplatelet effects. Xanthorrhizol reduced mRNA levels of MMP-2, MMP-8, NF-κB p65, and IL-1β in LPS-induced human gingival fibroblast-1 cells. The compound also has anti-inflammatory activities in H_2_O_2_-treated HT22 hippocampal cells and inhibited MAPK/activator protein-1 (AP-1) signaling pathways (Kim et al., 2018). In a study to evaluate the effects of standardized *C. zanthorrhiza* extract and its marker compound, xanthorrhizol, on hyperglycemia and inflammatory markers in high-fat diet–induced obese mice, xanthorrhizol was found to significantly inhibit inflammatory cytokine release, such as IL-1β, IL-6, TNF-α, and C-reactive protein (CRP), in the liver, muscle, and adipose tissue (Kim M-B et al., 2014).

#### Other Compounds

In addition to curcuminoids, sesquiterpenes like turmeronols have potential to suppress the immune response. Turmeronol A and turmeronol B from *C. longa* were tested on mouse macrophages (RAW 264,7 cells). Both turmeronols showed inhibitory of PGE_2_ and NO production as well as IL-6 and IL-1β at the mRNA and protein levels in LPS-induced cells. Turmeronols also inhibited the translocation of NF-κB from the cytoplasm into the nucleus (Okuda-Hanafusa et al., 2019). Curdione, a major compound of *C. aeruginosa*, has been proposed as a potential immunomodulatory agent. Molecular docking analysis revealed that there is a high probability of interaction between curdione and CD95 protein, as the replacement of native ligand. The docking score of curdione to the protein was lower than that of the native ligand. In addition, 1,8-cineol from *C. aeruginosa* also has a high docking score to CD95 protein, but not significant as compared to those of native ligand of CD95 (Anggriani et al., 2019). Isocurcumenol isolated from *C. zedoaria* did not demonstrate any significant toxicity on normal chicken embryo lymphocytes and fibroblast cells (Lakshmi et al., 2011). A recent study showed that five sesquiterpenoids (isoprocurcumenol, germacrone, curzerenone, curcumenol, and curcuzedoalide) from *C. zedoaria* demonstrated inhibitory activity on NO synthesis. Among the compounds, curcuzedoalide showed the highest inhibition. Further study showed that curcuzedoalide inhibited the expression of pro-inflammatory mediators (iNOS and COX-2) (Lee et al., 2019). Curcumin, dihydrocurcumin, tetrahydrodemethoxycurcumin, and tetrahydrobisdemethoxycurcumin in *C. zedoaria* enhanced the release of IL-4 and TNF–α, and inhibited the production of *ß*-hexosaminidase. *ß*-Hexosaminidase is a marker of antigen-IgE–mediated degranulation (Putri, 2014).

Isolated compounds from *C. zedoaria*, that is, epiprocurcumenol, procurcumenol, and 1,7-bis(4-hydroxyphenyl)-1,4,6-heptatrien-3-one, inhibited the production of TNF-α from LPS-activated macrophages (Jang, et al., 2001). These compounds, especially 1,7-bis(4-hydroxyphenyl)-1,4,6-heptatrien-3-one, were also found to exhibit strong inhibition against production of NO and expression of iNOS in activated macrophages (Jang et al., 2004). Demethoxycurcumin, bisdemethoxycurcumin, and 3-buten-2-one, 4-[(1R, 4aR, 8aR)-decahydro-5, 5, 8a-trimethyl-2-methylene-1-naphthalenyl]- (3E)-rel isolated from *C. manga* reduced the production of NO from LPS-stimulated RAW 264.7 cells. Among the compounds tested, demethoxycurcumin showed the highest NO inhibition (Kaewkroek et al., 2009). In an effort to elaborate the anti-inflammatory mechanism of compounds from *C. mangga* rhizomes, Kaewkroek et al. (2010) evaluated the anti-inflammatory effects of several compounds against production of PGE_2_ and NO from RAW 264.7 cells. These include demethoxycurcumin, bisdemethoxycurcumin, 15,16 bisnorlabda-8 (17),11-dien-13-one, and (E)-15,15-diethoxylabda-8 (17),12-dien-16-al. Of all the compounds tested, 11-dien-13-one (E)-15,15-diethoxylabda-8 (17),12-dien-16-al demonstrated the strongest NO inhibitory activity, while demethoxycurcumin displayed the strongest activity on PGE_2_ release. Moreover, investigation of mechanism at the transcriptional level showed that all the compounds reduced the mRNA expressions of COX-2 and iNOS, except 15,16 bisnorlabda-8 (17), 11-dien-13-one, which only downregulated the mRNA of iNOS.

Most of the studies on the bioactive secondary metabolites of *Curcuma* species were carried out at cellular and molecular levels on various immune cells to explore their effects on the release and expression of pro-inflammatory mediators *via* various signaling pathways, such as NF-κB, MAPKs, and other events. More in-depth studies to understand the underlying mechanisms using experimental *in vivo* animal models of immune-related disorders and elaborate bioavailability, preclinical pharmacokinetics, and toxicity studies are required before clinical trials can be pursued for development into immunomodulatory agents.

## Toxicological Studies

Systematic safety evaluations and toxicological investigations on *C. longa* and curcumin have indicated that they are nontoxic for human consumption, especially by oral administration. It is considered non-genotoxic, non-mutagenic, and generally recognized as safe. Several studies have indicated that oral administration of *C. longa* and curcumin in animals was safe without reproductive toxicity at certain doses. Clinical trials have indicated that the safe dose for human consumption was at an oral dose of 6 g/day for 4–7 weeks. In rare cases, minor side effects like gastrointestinal upsets may happen (Soleimani et al., 2018). A cheminformatics approach was used to predict toxicity, which includes human hepatotoxicity, rodent carcinogenicity, and bacterial mutagenicity of 200 chemical compounds found in *C. longa*. Of the compounds studied, 136 compounds were predicted as mutagenic, 184 were toxigenic, 64 were hepatotoxic, and 153 were carcinogenic. Interestingly, a dose-dependent hepatotoxicity may occur with curcumin and its derivatives. The study also predicted that few other constituents of *C. longa* are noncarcinogenic, non-mutagenic, non-hepatotoxic, and devoid of any side effects (Balaji and Chemakam, 2010).

Liju et al. (2013) reported the acute and sub-chronic toxicity studies of the essential oil of *C. longa* (EOCL). For the acute toxicity test, up to 5 g of EOCL per kg body weight was administered in a single dose to Wistar rats, while for the subacute toxicity study, the rats were administered with a daily oral administration at doses of 0.1, 0.25, and 0.5 g/kg for 13 weeks. The results indicated that the EOCL was nontoxic as there were no changes in body weight, and no mortality or adverse clinical signs during both acute and sub-chronic toxicity studies. The hepatic function was normal, and the biomarkers, alanine amino transferase (ALT), alkaline phosphatase (ALP), and aspartate aminotransferase (AST) remained unchanged in treated animals. There was no subacute toxicity as triglycerides, total cholesterol, serum electrolyte parameters, histopathology of tissues, and markers of renal function remained unchanged after 13 weeks of treatment with curcumin. There was no mutagenicity to *Salmonella typhimurium* up to 3 mg/plate. Oral administration of 1 g/kg body weight EOCL for 14 days did not produce any genotoxicity as there was no DNA damage and chromosome aberration or micronuclei in rat bone marrow cells (Mary et al., 2012).

The acute toxicity study of *C. zanthorrhiza* ethanol extract at 5000 mg/kg revealed that the extract did not show any toxicity signs such as salivation, sleeping, diarrhea, or lethargy in mice (Devaraj et al., 2010). The result was in accordance with a previous study which showed no toxicity sign was observed in rats after administration of *C. zanthorrhiza* ethanol extract at 2000 and 5000 mg/kg. During 14 days of observation, rats showed no clinical toxic signs, such as hypoactivity, hyperactivity, lethargy, dermatitis, anorexia, depression, and jaundice as well as no abnormalities in the kidney and liver (Rahim et al., 2014). Listyawati (2006) reported the chronic toxicity study of ethanol extract of *C. zanthorrhiza.* The extract at 50 mg/kg/day did not induce significant effects on spermatogenic and hematological changes (Listyawati, 2006). *C. zanthorrhiza* supplement at 2000 mg/kg showed no significant abnormalities on the lung, heart, liver, kidney, and stomach. The LD_50_ of *C. zanthorrhiza* supplement as hepatoprotective was greater than 5000 mg/kg bw (Arifin et al., 2020). Based on a clinical study on 30 healthy subjects, the administration of *C. zanthorrhiza* in combination with *Vitex trifolia* at doses of 1,500 and 4,500 mg/day for 14 days did not alter the liver and kidney function, while at a dose of 9000 mg/day, the administration altered the AST and serum creatinine values, indicating the extract affected the liver and kidney functions (Baroroh et al., 2011). An aqueous extract of *C. zanthorrhiza* at 2000 and 5000 mg/kg body weights also showed no toxicity in mice or rats. Xanthorhizol, the active constituent of *C. zanthorrhiza*, at a dose of 500 mg/kg did not cause mortality in mice (HMPC, 2014).

Our previous study reported the acute toxicity evaluation of ethanol extract of *C. mangga* rhizomes. Mice were administered with the extract at 500, 1000, 2000, and 5000 mg/kg body weights as a single dose, followed by 14 days of observation. Signs of toxicity were revealed as lethargy was observed after treatment with *C. mangga* extract at doses of 2000 and 5000 mg/kg body weight. Meanwhile, other signs of toxicity such as diarrhea, coma, and salivation were not recorded. In addition, the extract did not cause deleterious effect on mice body weight. Macroscopic examination of two main organs (liver and kidney) showed that the texture and color of both organs were comparable to those of normal group. *C. mangga* extract at the dose of 5000 mg/kg caused sinusoidal dilation in the liver and glomerular lesion in the right kidney; however, there was no lesion in the left kidney. The extract at the highest dose did not cause mortality; hence, it can be considered that the LD_50_ of *C. mangga* extract was estimated to be more than 5000 mg/kg body weight (Yuandani and Suwarso, 2017a).

The cytotoxicity evaluation of *C. aeruginosa* rhizomes on fibroblast test has been conducted by Yuliawati and Hestianah (2010). The results revealed that the extract at the concentrations ranging from 1 to 25 ppm was not toxic, as indicated by the percentage of cell viability ranging from 81.60 to 90.57% (Yuliawati and Hestinah, 2010). Sub-chronic toxicity evaluation of *C. aeruginosa* starch was conducted in Wistar rats. *C. aeruginosa* starch was administered daily for 90 days. The observation was performed for 90 days and followed until 120 days for satellite group to evaluate the reversible or irreversible effect. The hematological parameters were observed, these include leukocytes, hemoglobin, red blood cell (RBC), hematocrit, mean corpuscular hemoglobin concentration (MCHC), mean corpuscular hemoglobin (MCH), mean corpuscular volume (MCV), and platelet levels. The results indicated that there was no toxic effect on hematological parameters (Kusumarin et al., 2020). *C. aeruginosa* in combination with *Allium sativum*, *Terminalia bellirica*, and *Amomum compactum* has been evaluated for their safety. Acute toxicity evaluation has been performed according to the fixed-dose method of OECD guideline 420. The herbal formulation did not cause any toxic signs and symptoms. The were no abnormalities found on body weight gain, macroscopic and microscopic examinations as well as the relative organ weight after treatment with the herbal preparation at the doses of 300 and 2000 mg/kg body weight. Further study on sub-chronic toxicity showed that the extracts did not induce any physical toxic symptoms as well as abnormal weight gain and hematological parameters. Moreover, the herbal formulation at a dose of 4,032 mg/kg did not cause any toxic effects on the liver and kidney, which was indicated by the normal values of urea, creatinine, total protein, albumin, globulin, aspartate aminotransferase (AST) or glutamic oxaloacetic transaminase (GOT), and alanine aminotransferase (ALT). In addition, macroscopic and microscopic examinations showed that there were no toxic effect on all organs tested (Sholikhah et al., 2020).

The evaluation of acute toxicity study of the purified fraction of *C. zedoaria* revealed that the fraction at the dose of 41.6 and 35.7 mg/kg did not significantly alter the liver and kidney enzyme levels. The LD_50_ was 500 mg/kg bw (Lakshmi et al., 2011). Furthermore, *C. zedoaria* ethanol extract at 150 mg/kg/day revealed a significant reduction of RBC, Hb level, and spermatozoa quality in chronic toxicity evaluation (Listyawati, 2006). The essential oil of *C. zedoaria* at 100 or 200 mg/kg revealed weight loss, and abnormal hematological and biochemical changes on dams and embryos in GD17 pregnant rats. The toxicity mechanism may be related to placental calcification in angiogenesis (Zhou et al., 2013). Sudeepthi et al. (2014) reported the safety evaluation of *C. amada* rhizomes in short-term treatment. The acetone extract of *C. amada* (500–2000 mg/kg) was administered to the test animals. The results indicated that the highest dose tested did not cause mortality (Sudeepthi et al., 2014).

## Conclusion and Future Directions

In the last 20 years, many plants of the genus *Curcuma* especially *C. longa*, *C. zanthorrhiza*, *C. amada*, *C. mangga*, *C. aeruginosa*, and *C. zedoaria* and some of their bioactive compounds have been investigated for their immunomodulating effects on the immune system. Most of the studies were *in vitro* and *in vivo* and only a few of the preclinical studies have progressed into clinical studies. The up-to-date literature gathered indicated that the immunological investigations on the plant extracts were mainly preliminary with little mechanistic studies. Most of the studies were on the crude extracts of the rhizomes. The extracts were not appropriately characterized chemically or standardized to the bioactive marker compounds which were responsible for the activity. The contributions of the chemical constituents of the plant to the bioactivities were not clearly correlated and identified. It is necessary for the immunomodulatory activity studies of the plant extracts to be accompanied with analyses of their bioactive compounds and identification of the chemical markers for standardization purposes. The extracts used in these studies should be quantitative and qualitative analyzed by using validated analytical methods. Some of the bioactive compounds especially the curcuminoids (curcumin, demethoxycurcumin and bisdemethoxycurcumin) and some sesquiterpenoids have been isolated from the extracts and their mechanistic effects in modulating the immune system have been determined. However, more mechanistic studies should be carried out for in depth understanding of the modulating effects of the plant samples on the innate and adaptive immune system. Of all the *Curcuma* species investigated, the immunomodulatory effects of *C. longa* and its major compound, curcumin, were the most studied. Their modulatory effects on various signaling pathways at molecular level have been reported. However, extensive molecular work on the other *Curcuma* species need to be carried out. Despite the regulatory requirements for clinical studies and sufficient data have not been generated on preclinical testing, there were already reports on a few unsystematic case studies to evaluate the immunomodulating properties of *Curcuma* species in human. Some clinical trials have been conducted on *C. zanthorrhiza* but they were unsystematic and not well designed. There was also lack of sufficient preclinical data and the extracts were not appropriately standardized. For clinical studies, sufficient preclinical testing should be generated using standardized extracts, which include bioavailability, pharmacokinetic and toxicological studies, before they can be subjected to clinical studies. Of all the bioactive metabolites of Curcuma species, only curcumin is undergoing extensive clinical trials for its anti-inflammatory properties and potential use as an adjuvant in the treatment of cancer. Curcumin has showed significant ability to modulate the immune response in experimental and clinical studies. However, more systematic and operationally thorough controlled randomized trials are needed to prove its safety and efficacy for human use. Other compounds from *Curcuma* species such as xanthorrhizol, curdione, curcuzedoalide, isoprocurcumenol and turmeronols have also been reported to modulate various lineages of immune response. More in depth studies including elaborate bioavailability, preclinical pharmacokinetics and toxicity studies are required to understand the underlying mechanisms and safety level before clinical trials can be pursued for development into potent and safe immunomodulatory agents.

## Glossary

ADMET: absorption, distribution, metabolism, elimination, and toxicity
ALP: alkaline phosphatase
ALT: alanine amino transferase
AP-1: activator protein-1
AMPs: antimicrobial peptides
APC: antigen-presenting cells
AST: aspartate aminotransferase
C5aR: complement C5a receptor
CL: chemiluminescence
CMS: chronic mild stress
CO_2_: carbon dioxide
CRP: C-reactive protein
DTH: delayed hypersensitivity
EOCL: essential oil of C. longa
fMLP: formyl methionyl-leucyl-phenylalanine;
fMLPR: formyl-methionyl-leucyl-phenylalanine receptor
GOT: glutamic oxaloacetic transaminase
HFD-: high-fat diet-
HOCl: hypochlorous acid
HUVEC: human umbilical vein endothelial cells
IBV: infectious bronchitis virus
IBD: inflammatory bowel diseases
IgM: immunoglobulin M
IL-12: Interleukin 12
IL-6: interleukin-6
IL-1β: interleukin-1β
iNOS: inducible nitric oxide synthase
ISI: Institute for Scientific Information
IκB-α: I kappa B alpha
LPS: lipopolysaccharide
MAPKs: mitogen-activated protein kinases
MCHC: mean corpuscular hemoglobin concentration
MCH: mean corpuscular hemoglobin
MCP1: monocyte chemoattractant protein-1
MCV: mean corpuscular volume
MIP1α: macrophage inflammatory protein-1α
MHC: major histocompatibility complex
MPO: myeloperoxidase
NO: nitric oxide
NF-ĸB: nuclear factor-kappa B
PAFR: platelet-activating factor receptor
PGE2: prostaglandin E2
PHA-P: phytohemagglutinin-P
PMNs: polymorphonuclear cells
PMA: phorbol 12-myristate 13-acetate
PUFAs: polyunsaturated fatty acids
ROS: reactive oxygen species
SLE: systemic lupus erythematosus
sRBCs: sheep red blood cells
T1D: type 1 diabetes
TLC: total leukocytes number
TNF-α: tumor necrosis factor-alpha.
